# Global expression patterns of *R*-genes in tomato and potato

**DOI:** 10.3389/fpls.2023.1216795

**Published:** 2023-10-27

**Authors:** Janina K. von Dahlen, Kerstin Schulz, Jessica Nicolai, Laura E. Rose

**Affiliations:** ^1^ Institute of Population Genetics, Heinrich-Heine University Duesseldorf, Duesseldorf, Germany; ^2^ iGRAD-Plant Graduate School, Heinrich-Heine University Duesseldorf, Duesseldorf, Germany; ^3^ Ceplas, Cluster of Excellence in Plant Sciences, Heinrich-Heine University Duesseldorf, Duesseldorf, Germany

**Keywords:** resistance genes, immune system, plant-pathogen interactions, Solanaceae, NBS-LRRs, NRCs, gene regulation

## Abstract

**Introduction:**

As key-players of plant immunity, the proteins encoded by resistance genes (*R*-genes) recognize pathogens and initiate pathogen-specific defense responses. The expression of some *R*-genes carry fitness costs and therefore inducible immune responses are likely advantageous. To what degree inducible resistance driven by *R*-genes is triggered by pathogen infection is currently an open question.

**Methods:**

In this study we analyzed the expression of 940 *R*-genes of tomato and potato across 315 transcriptome libraries to investigate how interspecific interactions with microbes influence *R*-gene expression in plants.

**Results:**

We found that most *R*-genes are expressed at a low level. A small subset of *R*-genes had moderate to high levels of expression and were expressed across many independent libraries, irrespective of infection status. These *R*-genes include members of the class of genes called NRCs (NLR required for cell death). Approximately 10% of all *R*-genes were differentially expressed during infection and this included both up- and down-regulation. One factor associated with the large differences in *R*-gene expression was host tissue, reflecting a considerable degree of tissue-specific transcriptional regulation of this class of genes.

**Discussion:**

These results call into question the widespread view that *R*-gene expression is induced upon pathogen attack. Instead, a small core set of *R*-genes is constitutively expressed, imparting upon the plant a ready-to-detect and defend status.

## Introduction

Plants are constantly in contact with an array of microbes; some of which may harm the plant, some of which may benefit the plant. A challenge for every species at the outset of an encounter with a potential pathogen is to initiate an appropriate, coordinated cellular and organismal-level response. The plant immune system works to restrict the pathogen’s ability to damage the host. Key-players of plant immunity are resistance genes (*R*-genes; reviewed in Jones and Dangl, 2006). Their protein products, R-proteins, recognize secreted pathogen-specific effectors, which may encode proteins, peptides or other molecules. These molecules interfere with the host’s physiology, including the immune system. In some cases, pathogen molecules manipulate host gene expression or inactivate host secreted proteolytic enzymes (Allen et al., 2004; Song et al., 2009; Fabro et al., 2011). *R*-gene mediated recognition can involve direct recognition through the binding of a pathogen effector by a corresponding R-protein or via indirect recognition by monitoring effector-altered endogenous plant proteins (Jones and Dangl, 2006; Kourelis and van der Hoorn, 2018). R-proteins are the activators of a powerful, pathogen-specific immune response, which often includes transcriptional re-programming (Glazebrook, 2005; Tsuda and Katagiri, 2010). Recently it has been shown that ZAR1, encoded by an *R*-gene, is the basis of a structure called the resistosome and is directly involved in initiating the hypersensitive resistance response (Wang et al., 2019a). When activated, ZAR1 forms a pore within the cell wall that causes the cell to leak and leads to cell death. Given the diversity of the potential antagonistic interspecific encounters, it is clear that the range of recognition specificities and the ability to orchestrate appropriate downstream responses cannot be achieved by a limited number of host defense proteins. Not surprisingly, *R*-genes in plants are encoded by large multi-gene families (Jupe et al., 2013; Andolfo et al., 2014; Gao et al., 2018; Lee and Chae, 2020). The largest class of *R*-genes is the NBS-LRR class, which stands for Nucleotide Binding Sites (NBS) and Leucine Rich Repeats (LRRs; Jones and Dangl, 2006). The recognition of effectors is typically mediated by the LRR-domain, while the NBS-domain functions as a molecular switch, activating downstream components that initiate plant defense (McHale et al., 2006). Other classes of *R*-genes encode enzymatic proteins and lack NBS/LRR domains (e.g. *Hm1*, *Pto*, *Rpg1*; reviewed in Gururani et al., 2012).

A tight regulatory system controls the expression of *R*-genes (Stokes et al., 2002; Holt et al., 2005; Li et al., 2007; Huot et al., 2014). One layer of regulation is mediated by transcription factors which alter gene expression by binding to upstream elements of genes (reviewed in Latchman, 1997). Transcription factors can enhance or repress the expression of *R*-genes (e.g. ethylene-responsive factor ERF; Chakravarthy et al., 2003). Another mode of gene regulation is RNA silencing, a sequence-specific system that uses small non-coding RNAs (sRNAs) to repress gene expression (reviewed in Baulcombe, 2004). These sRNAs are guided via sequence-complementarity to target mRNAs which, together with Argonaute proteins, degrade or inhibit translation of mRNA transcripts (Baulcombe, 2004). One example of such sRNA-mediated gene suppression of *R-*genes is the microRNA (miRNA) superfamily miR482/2118 (Shivaprasad et al., 2012; de Vries et al., 2015; de Vries et al., 2018). Another mode of transcriptional regulation is mediated through alternative splicing. In the context of *R-*genes, it has been shown that different splice variants of the same *R*-gene can lead to the expression of distinct R-proteins which underlie different resistance phenotypes (e.g. splicing variants NAT and NRT of the resistance gene *N*; Yang et al., 2014).

The Solanaceae plant family harbors many economically important crops including potato, tomato, eggplant, pepper and tobacco. As a chief non-cereal crop, potato cultivation yielded 487 million tons in 2017. Due to the economic significance of species in this plant family, a large body of data is available regarding the genetic basis of pathogen resistance. This includes well-described resistance gene repertoires and large-scale transcriptome studies of these species from a range of tissues, time points, cultivars and pathogen treatments. In this study, we analyzed the expression profiles of 940 *R*-genes from tomato and potato using 315 transcriptomes with and without pathogen treatment.

We determined that the majority of *R*-genes in tomato and potato are constitutively expressed at a low level, irrespective of infection status. Based on our analyses, we could define a core set of *R*-genes which are expressed in greater than 90% of all libraries in each species. For tomato, the core set comprises 7.7% of the *R*-genes; in potato 16.6% of the *R*-genes belong to the core set. Members of the core are well known *R*-genes such as *EDS1* and *Pto* as well as NRC2, NRC3 and NRC4, powerful activators of immunity. Analysis of similarity (ANOSIM) based on relative gene expression showed that the two main factors that explain variation in *R*-gene expression are tissue type and “BioProject”. A BioProject is defined by NCBI as a collection of biological data related to a single initiative, originating from a single organization or from a consortium. In our ANOSIM analysis, infection status and infection time were not associated with significant differences in *R*-gene expression. In an independent analysis based on differential gene expression of paired libraries, we determined that 11.9% of *R*-genes in tomato and 8.6% in potato are differentially expressed in the presence of a microbe treatment. In potato, the same proportion of genes are up-regulated or down-regulated, while in tomato a larger proportion is up-regulated following treatment with microbes. The factors BioProject, tissue type or distinction between treatment with beneficial or pathogenic microbes were not associated with differential gene expression. These results indicate that plants express a core set of *R*-genes, ensuring that they are in a permanent ready-to-defend status. We find little evidence that this class of genes responds with large-scale, shared transcriptional reprogramming following exposure to pathogenic microbes.

## Material and methods

### Transcriptome data sets

A total of 315 transcriptome datasets of tomato (Zouari et al., 2014; Du et al., 2015; Barad et al., 2017; Sarkar et al., 2017; Sugimura and Saito, 2017; Xue et al., 2017; Yang et al., 2017; Zheng et al., 2017; Chen et al., 2018; Shukla et al., 2018; Fawke et al., 2019; Pesti et al., 2019; Wang et al., 2019b) and potato (Goyer et al., 2015; Zuluaga et al., 2015; Dees et al., 2016; Gao and Bradeen, 2016; Kochetov et al., 2017; Levy et al., 2017; Li et al., 2017; Lysøe et al., 2017; Hao et al., 2018; Kumar et al., 2018) were obtained from the Sequence Read Archive (**Figure S1**). These studies included treatments with potentially beneficial organisms (arbuscular mycorrhizal fungi (AMF) and biocontrol agents) as well as detrimental organisms (pathogenic bacteria, nematodes, fungi, viruses, viroids, insects and oomycetes). Only studies with at least one mock treatment were included. The collected tissues included roots, stems, leaves, fruits and tubers. The time points of sampling after infection range from 0 days post-infection (dpi) up to 42 dpi or until the end of the host’s life cycle (**Figure S1**). Approximately 20% of all tomato and potato cultivars were denoted as resistant to the applied pathogens.

### *R*-gene data set

The lists of the *R*-gene repertoires of *S. lycopersicum* and *S. tuberosum* were retrieved from Jupe et al. (2013). *R*-genes were classified as “full-length” NBS-LRRs if they contained both NBS and LRR domains as identified using InterPro (Mitchell et al., 2019). A slightly modified pipeline as described by Jupe et al. (2013) was used to verify their novel *R*-genes (**Figure S2**). These novel *R*-genes were designated by the authors as *R gene discovery consortium* (*RDC*) genes. Using AUGUSTUS (version 3.3.1), a gene-prediction tool developed by Stanke et al. (2008), we analyzed these *RDC* genes for coding regions and searched for NBS and LRR domains using InterPro. *RDCs* were classified as true *R*-genes if they possessed a coding region and an NBS-LRR domain. Otherwise they were excluded from further analysis. In cases in which multiple splice variants were identified, the longest splice variant was analyzed. The well-established *R*-genes *Pto* (Martin et al., 1993) and *EDS1* (Hu et al., 2005) from tomato were included in the dataset. In total, the expression patterns of 359 *R*-genes of tomato and 581 *R*-genes of potato were analyzed.

### Identification of physical clusters of *R*-genes

*R*-genes were classified as belonging to a cluster when more than one *R*-gene was located in a region of 200 kilobases (kb) on a chromosome (Van de Weyer et al., 2019). Since Jupe et al. (2013) performed their analysis on an earlier release of the tomato genome assembly (ITAG2.4 release, Tomato Genome Consortium, 2012), the positions of all tomato *R*-genes had to be re-defined (**Table S1**). Positions of *RDCs* were verified using Blastn v2.6.0 (Altschul et al., 1990; Camacho et al., 2009) against the tomato (ITAG4.0; Hosmani et al., 2019) and potato genomes (PGSC_DM_v4.03; Potato Genome Sequencing Consortium, 2011; **Table S1**). All *R*-genes without defined chromosomal positions (39 genes in tomato) were classified as *R*-genes with unknown clustering.

### miRNA targeting prediction

To predict potential regulation of *R*-genes by the miR482-superfamily (de Vries et al., 2015), we used psRNATarget (release 2017; Dai et al., 2018). We used the coding sequence (CDS) of our *R*-genes as the target library. To ensure a low rate of false-positives, the maximum expectation was set to ≤3, since higher expectation values represent less likely mRNA/miRNA interactions. We evaluated the likelihood of an *R*-gene being targeted by the miR482 superfamily and whether the *R*-gene encoded a full length NBS-LRR and or belonged to a *R*-gene cluster using a chi-square test (Greenwood and Nikulin, 1996).

### Calculation of transcript abundance using Kallisto

The program Kallisto (v.0.46.0) was used to estimate the relative expression of genes in tomato and potato (Bray et al., 2016). As a first step, the raw sequence reads were compared to the transcript sequences. This step in Kallisto is designated as the pseudoalignment step. To improve the quality of the pseudoalignment, low-quality reads and adapters were removed from the transcriptomes using Trimmomatic under the following settings: seed mismatch = 2; palindrome clip threshold = 30; simple clip threshold = 10; LEADING = 3; TRAILING = 3; SLIDINGWINDOW= 4:15; MINLEN =36 (Bolger et al., 2014; **Figure S2**). Subsequent quality controls were performed using FastQC (Andrews, 2010). As Kallisto requires information on fragment length for single-end sequenced transcriptomes, the fragment length denoted by the authors was used. If this information was not available, the recommended fragment length of the reported RNA isolation kit was used. The standard deviation was set to ±17.5 bp. Kallisto indices (used for generating the pseudoalignments) were based on the tomato ITAG4.0 and the potato PGSC_DM_v4.03 genome releases. *R*-genes missing from the current genome releases were manually added to the list of transcripts (indices in Kallisto). Transcript abundance was calculated as transcripts per million (TPM; Wagner et al., 2012). We chose to use TPM since it normalizes the transcript abundance for gene length and library size, making TPM values comparable across experiments. Genes for which the TPM values were less than 1 were treated as “off” and for these genes, TPM was set to zero. All scripts and settings used for these analyses are available at the following site: https://github.com/LauraERose/LargeScaleTranscriptomeAnalysis.

### Verification of gene expression using qRT-PCR

To verify the overall consistency of our estimated TPM values in this metaanalysis, we performed qRT-PCR on twelve NBS-LRR-genes and three reference genes (de Vries et al., 2018). We evaluated the expression of these fifteen genes over six time points on the Moneymaker cultivar inoculated with *Phytophthora infestans* (*P. infestans*) isolate IPO-C. Three replicates were studied at each sampling time point and treatment type. Additional details of this experiment are reported in de Vries et al., 2018. The Bioproject L (PRJNA487149) from Fawke et al., 2019 is the most similar in design to our 2018 study, since that project sampled transcriptomes from leaves of the tomato cultivar ‘MicroTom’ inoculated with *P. infestans* isolate 88069. We evaluated the consistency between the average TPM of these 15 genes from Fawke et al. with our estimated Cq values at 72 hours post infection, the single overlapping timepoint between both data sets.

### Comparison of gene expression across gene sets

To compare the mean relative expression between *R*-genes (*R*-gene set size for tomato = 359 and for potato = 581) and non-*R*-genes (the rest of the genome) we generated 100 replicate datasets for each transcriptome by sampling the TPM values of 359 random genes from tomato and 581 random genes from potato. The average TPM of all expressed genes was calculated for each replicate dataset. To compare expression values, four reference genes were used: ubiquitin (*Solyc09g018730.4.1*) and actin4 (*Solyc04g011500.3.1*) for tomato (Müller et al., 2015) an importin subunit (*PGSC0003DMG400007289*) and elongation factor-1 (*PGSC0003DMG400023270*) for potato (Mariot et al., 2015; Tang et al., 2017). TPM values were tested for normality using the Anderson-Darling (>5000 data points; Thode, 2002) or Shapiro test (< 5000 data points; Shapiro and Wilk, 1965) and for equal variances using the test from Kendall (1938). Significant differences in expression were identified using a Mann-Whitney-U test (Mann and Whitney, 1947) for non-normally distributed data or a two-sample t-test for normally distributed data.

We visualized *R*-gene expression using heatmaps created in R (v. 3.6.1). Genes were classified as off (if TPM < 1) or on (if TPM ≥1). In the heatmaps, libraries were clustered by similarity in patterns of expression between libraries and *R*-genes were sorted by the number of libraries expressing the corresponding gene. Correlations between 1) the total number of expressed *R*-genes and the total number of expressed genes, 2) the total number of expressed genes and the number of pseudo-aligned reads, as well as 3) the number of libraries in which an *R*-gene was expressed and the average level of expression of each *R*-gene were performed using a Spearman’s rank correlation test (Hollander et al., 2013).

To investigate the extent to which expression patterns of *R*-genes were similar to wild close relatives of tomatoes, we evaluated additional transcriptomes of four wild tomato species: *S. peruvianum*, *S. chilense*, *S. ochranthum*, and *S. lycopersicoides* (Beddows et al., 2017). A subset of *R*-genes was further analyzed for their patterns of sequence variation within and between these wild species. Standard population genetic parameters including intraspecific variation (π) and interspecific divergence (K) were estimated using DNaSP v. 5.10 (Librado and Rozas, 2009).

### Analysis of differences in expression

To identify the factors associated with differences in expression of *R*-genes across transcriptomes, we performed an ANOSIM in Primer 7.0.13 (PRIMER-e; **Figure S2**). ANOSIM is a non-parametric statistical test similar to ANOVA. The starting point of the analysis is a pairwise dissimilarity matrix. In our case, the dissimilarity matrix was computed as follows: First the TPM values for each gene within each transcriptome were LOG (x+1) transformed. On the basis of these transformed TPM values, the dissimilarity in gene expression patterns between transcriptomes were calculated based on Euclidean distances. Ranking was applied to the distance matrix. The two libraries from potato (SRR6511453 and ERR791944) with exceptionally low expression of the entire *R*-gene repertoire were excluded in these analyses.

To determine if gene expression is more similar within groups than between groups (for example when groups are defined by infection status or tissue type) the R test statistic value was calculated. The R values can range from -1 to 1, with larger values corresponding to greater differences between groups. Statistical significance is calculated through permutation of the group labels and recalculation of the R value for each replicate. In our case, 999 permutations were generated. The following factors were evaluated: BioProject, tissue type, type of treatment, specific treatment organism, life cycle of the organism, type/kingdom of the organism, susceptible vs. resistant cultivar, relative sequencing depth, paired- or single-end sequencing and days post infection. The ANOSIM analysis was also applied to the differential gene expression data (see below).

### Differential expression analysis

Differentially expressed genes between microbe treatments and mock treatments were identified using Sleuth (Pimentel et al., 2017; **Figure S2**). The p-values were adjusted using the Benjamini-Hochberg correction (FDR ≤ 0.05; Benjamini and Hochberg, 1995). Since Sleuth relies on replicates within treatments, BioProjects without replicates were removed from this part of analysis. We evaluated differences between i) *R-genes* and all genes, ii) proportion of up- versus down-regulation iii) average absolute fold changes.

## Results

### Large scale patterns of *R*-gene expression

In total we analyzed 7.78 x10^9^ raw reads from 315 transcriptomes of tomato and potato of which 5.58 x10^9^ could be uniquely assigned to a transcript from tomato/potato (average proportion of assigned reads: 77.3% for tomato and 66.8% for potato; **Figure S3**). Both mock-inoculated plants as well as plants inoculated with pathogenic and beneficial organisms were investigated. In total, 359 *R*-genes from tomato and 581 from potato were examined for their expression levels and fold changes. In tomato, 62.1% of all *R*-genes possessed NBS- and an LRR-domains and in potato 89.2% did (**Figure S4**, **Table S1**). A large majority of the *R*-genes of both species formed physical clusters meaning that two or more *R*-genes were found in a span of 200kb along the chromosome (62.6% in tomato; 83.1% in potato).

Since the miR482-superfamily is a known regulator of NBS-LRR expression (Shivaprasad et al., 2012; de Vries et al., 2015; de Vries et al., 2018), we evaluated the targeting probability by members of the miR482 gene family for each *R*-gene. In tomato 17.6% of all *R*-genes were predicted to be targeted by the miR482-superfamily, while in potato 28.6% were predicted to be targeted (**Figure S4**, **Table S1**). It has previously been shown that miR482-members regulate *R*-genes by reverse-complementary binding to the mRNA region encoding NBS-domains (Shivaprasad et al., 2012). Full length *R*-genes were more likely to be predicted to be regulated by the miR482-superfamily compared to partial length NBS-LRR genes (χ^2^_tomato_ = 21.32, p-value < 0.001; χ^2^_potato_ = 14.69, p-value < 0.001; **Figure S4**; **Table S2**).

### Most *R*-genes show consistently low expression, both in the presence and absence of pathogens

**Figure 1:**
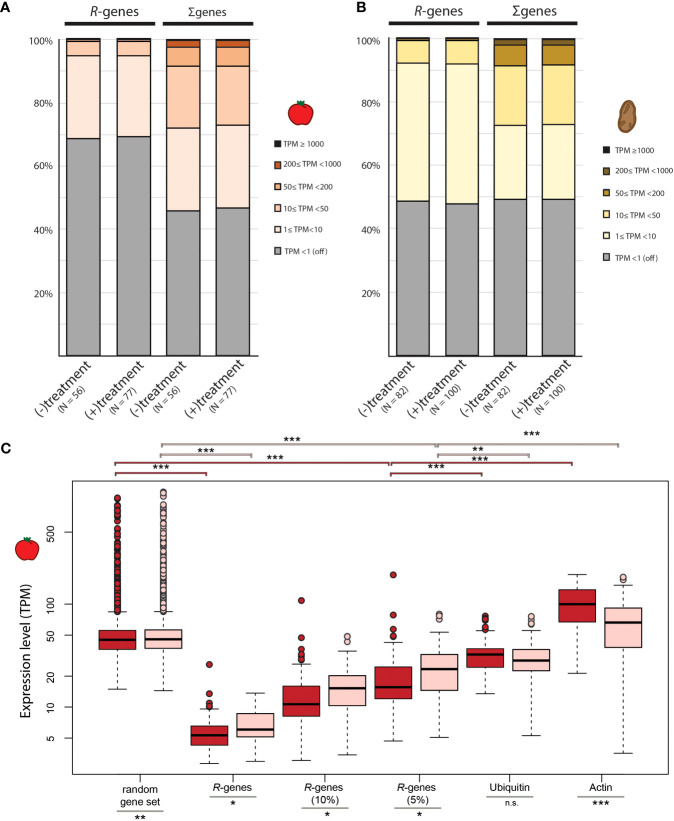
Comparison of relative gene expression in tomato and potato. Relative expression of *R*-genes compared to the rest of the genes in the genome for **(A)** tomato and **(B)** potato. Each gene was assigned to 1 of 6 expression categories based on TPM. **(C)** Mean TPM for gene sets in libraries from mock-treated plants (dark red) and plants treated with organisms (light red). Random gene subsets were created by sampling 359 genes randomly (matching the number of *R*-genes in tomato) from each tomato library and calculating the mean TPM of these 359 genes across each library. Overall 100 random gene sets (containing different sets of 359 genes) per library were created and the average TPM across the 100 replicates is displayed in the box plot format. The distribution of gene expression (TPM values) for the top 10% and 5% of the set of *R*-genes in each library are displayed as well as the mean TPM for two reference genes (ubiquitin and actin). The midline of each box is the median, boxes extend from the 25th to the 75th percentile, and the dots are outliers. Pairwise differences were computed using either a Mann-Whitney-U test for non-normally distributed data or a two-sample t-test for normally distributed data: n.s. = not significantly different; * p-value <0.05; ** p-value <0.01; *** p-value <0.001.

Proteins encoded by *R*-genes act as key regulators of plant immunity by recognizing plant pathogens and activating the plant immune response. However, the existence of growth-defense trade-offs implies that the constitutive expression of *R*-genes in the absence of pathogens might be costly (reviewed in Brown and Rant, 2013; Vos et al., 2013). In our study, a large proportion of the *R*-gene repertoire in tomato (67.6% ± 13.8%) is not expressed in a given library (or is below the threshold of detection) whether or not the plant was treated with an interacting organism (**Figure 1A**). A smaller proportion (~ 46%) of the non-*R*-genes are “off” or below the threshold of detection in tomato (**Figure 1A**). For potato, the proportion of the *R*-gene repertoire that is not expressed is 49.3% (± 11.7%); this is nearly equal to the proportion of genes that are not expressed in the rest of the genome (**Figure 1B**).

In both species, the average TPM of *R*-genes per library is significantly lower than the average TPM of an equal number of randomly selected genes per library (p-value <0.001; **Figures 1C**, **S5**). Of the *R*-genes that are expressed, most are expressed at very low levels within each library (between 1 and 10 TPM). Approximately one quarter of *R*-genes in tomato (25.9% ± 8.4%) and 44.0% (± 9.8%) of *R*-genes in potato are expressed at this level. Less than 1% of the *R*-genes fall into the medium (50≤ TPM <200) or high (200≤ TPM <1000) expression classes, a scant proportion for these two expression classes compared to non-*R*-genes (**Figures 1A, B**).

The distribution of the expression classes for *R*-genes varies greatly across libraries (**Figure S6**). For example, 88.3% of *R*-genes are not expressed in library SRR7073605, while in library SRR442353, 47.0% are not expressed (**Figure S6**). Although the relative transcript abundance of a few *R*-genes can be high, the average TPM of the top 10% (or even the top 5%) is still well below the average TPM across all other genes in the genome for a given library (**Figure 1C**; **S5**; p-value_tomato_ <0.001; p-value_potato_ <0.001). Taken together, most *R*-genes are typically expressed at low to extremely low levels across libraries.

In our study, the overall distribution of expression classes of *R*-genes is similar between plants treated with interaction partners versus untreated controls (**Figures 1A, B**, **S6**). However, conditioning on only the expressed *R*-genes in each individual library, the average expression level (measured as TPM) of these expressed *R*-genes is significantly higher in tomato plants treated with microbes compared to mock treated controls (p-value <0.05; **Figure 1C**). This effect was specific for treatment with pathogenic organisms: We observed that the average TPM-values for expressed *R*-genes (TPM >1) was higher in tomato plants exposed to pathogenic organisms compared to plants exposed to beneficial microbes (p-value <0.001; **Figure S7A**). In contrast, in potato no difference in the average expression of *R*-genes between treated and untreated plants, nor between the types of treatments (pathogenic versus beneficial) could be detected (p-value >0.05; **Figure S5**; p-value = 0.58; **Figure S7B**).

### Some *R*-genes are consistently expressed across libraries

**Figure 2:**
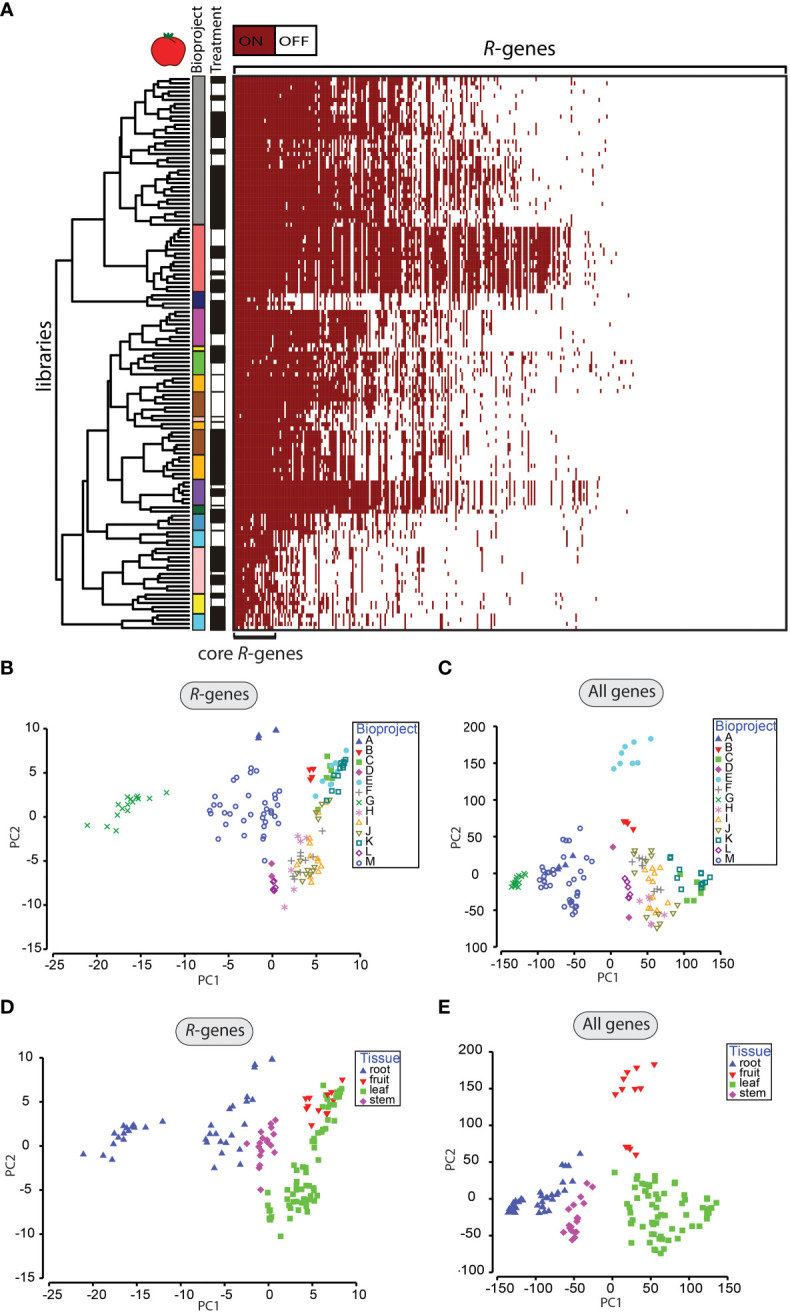
Patterns of *R*-gene expression in tomato. **(A)** Heatmap of *R*-gene expression (359 genes) from tomato (133 libraries). Genes were classified as off/white (if TPM <1) and on/red (if TPM ≥1). Libraries were clustered by similarity in patterns of expression between libraries. *R*-genes were sorted by the number of libraries expressing the corresponding *R*-gene from highest (left) to lowest (right). Assignments to individual BioProjects are indicated by different colors in the first vertical column next to the dendrogram. The treatment status of the libraries with mock-treated (white) or treated with an organism (black) is displayed in the 2nd vertical column next to the dendrogram. **(B-E)** Principal component analysis of gene expression of *R*-genes **(B, D)** and all genes **(C, E)**. Samples are labeled by the BioProject **(B, C)** or by the tissue type **(D, E)**. Clustered groups indicate higher levels of similarity in gene expression.

We observed that some *R*-genes were expressed (TPM ≥1) under both challenged and unchallenged conditions. Therefore, the question arose if these *R*-genes represent a “core set” of expressed *R*-genes across all libraries. Approximately 7.7% of all *R*-genes in tomato are expressed in > 90% of all analyzed libraries (**Figure 2A**). In potato, 16.6% of all *R*-genes were expressed in >90% of all libraries (**Figure S8A**). Among these expressed “core” *R*-genes in tomato are *EDS1*, *Pto*, NRC2, NRC3 and NRC4. Wu et al. (2017) identified these NRCs as part of a complex network in Solanaceae in which the NRCs (*Solyc10g047320*, *Solyc05g009630*, *Solyc04g007070*) interact with NBS-LRR sensors to activate resistance.

We evaluated whether this set of expressed “core” *R*-genes shared other characteristics. We found that the mean TPM-value of an *R*-gene within a library was positively correlated with expression breadth as defined as the number of libraries in which it was expressed (correlation factor rho_tomato_ = 0.39, p-value <0.000, rho_potato_ = 0.47, p-value < 0.000, **Figures S9A, B**). Therefore, this set of expressed “core” *R*-genes has both higher relative expression within a library and broader expression across libraries than non-core *R*-genes.

The total number of *R*-genes expressed per library varied from 27 to 191 in tomato, with a mean proportion of ~30% of *R*-genes expressed in a given library (**Table S3**; **Figure S10A**). For potato, the number of *R*-genes expressed per library ranged from 1 to 421 *R*-genes, with a mean proportion of 50.8% of the *R*-genes expressed in a given library (**Table S4**; **Figure S10B**). We also evaluated whether the proportion of expressed *R*-genes correlated with the total number of expressed genes in a given library. In both potato and tomato, libraries in which a larger number of genes were expressed also had a higher proportion of expressed *R*-genes (rho_tomato_ = 0.84, rho_potato_ = 0.71, p-value <0.000; **Figures S9C, D**). We investigated how the distribution of the proportion of *R*-genes expressed correlated with the proportion of assigned reads (as a proxy for sequencing quality). Overall, we detected a weak positive correlation between both factors (rho_tomato_ = 0.39, rho_potato_ = 0.42, p-value <0.001; **Figures S9E, F**).

### Factors associated with variation in *R*-gene expression across libraries are BioProject and tissue type

We applied an ANOSIM method to evaluate which factors were associated with variation in *R*-gene expression across the libraries (**Table 1**; **Table S5**; **Table S6**). In the ANOSIM analysis, higher R-values indicate a larger influence of a factor on the patterns of gene expression. The factor with the highest R-value for *R*-genes was BioProject (R-value_tomato_ = 0.876, p-value <0.001; R-value_potato_ = 0.928, p-value <0.001, **Table 1**). Differentiation by BioProject is also apparent in the principal component analysis (PCA, **Figures 2B, C**, **Figures S8B, C**). Libraries clustering closer together in the PCA indicate those with more similar expression patterns. In this study, the factor BioProject corresponds to the set of libraries submitted by a single lab group. In total, 13 BioProjects for tomato were studied and 12 BioProjects for potato. The number of libraries submitted as part of a BioProject ranged from as low as two and up to 36. In some cases, BioProjects sampled only a single tissue type; other BioProjects sampled multiple tissue types. Most BioProjects focused only on a single potato or tomato cultivar. Individual BioProjects typically included treatment with one main (micro-)organism, except for a handful which studied treatments with two or more organisms. Due to the diversity of projects in terms of plant genotypes, type of organismal challenge and time of sampling and since the sampling was not based on a nested design, the large effect of the BioProject is not unexpected. However, the value of such a meta-analysis is that robust and consistent patterns of gene expression that do emerge from this study, in the face of a large amount of experimental variation across labs, are likely to be highly reliable because a wide range of sampling conditions were included (different lab conditions, different cultivars, different time of sampling, different treatments, etc.). Furthermore, this type of analysis can be used to identify key experiments that are missing (such as tissue type, time of sampling, cultivar, or pathogen) that if included could provide the necessary cross-lab validation of patterns.

**Table 1:** ANOSIM analysis of (*R*-)gene expression.

Organism	Factor	All genes		*R*-genes	
		R-value	p-value	R-value	p-value
**Tomato**	**BioProject** (A through M)	0.959	0.1%	0.867	0.1%
	**Tissue type** (roots, fruit, leaf, stem)	0.689	0.1%	0.527	0.1%
	**Paired- or single-end sequencing**	0.495	0.1%	0.358	0.1%
	**Days post infection** (0 days till end of life cycle of the plant)	0.361	0.1%	0.408	0.1%
	**Relative sequencing depth** (5 categories from low to high)	0.312	0.1%	0.297	0.1%
	**Specific treatment organism** (14 types)	0.203	0.1%	0.264	0.1%
	**Life cycle of the organism** (5 types)	0.152	0.1%	0.189	0.2%
	**Type/Kingdom of the organism** (6 kingdoms)	0.14	0.1%	0.242	0.1%
	Susceptible vs resistant cultivar*	0.103	0.1%	0.079	0.7%
	**Type of treatment** (3 treatments)	0.068	0.2%	0.05	2.7%
**Potato**	**BioProject** (A through L)	0.92	0.1%	0.928	0.1%
	**Tissue type** (tuber, root, leaf)	0.766	0.1%	0.758	0.1%
	Tissue type II** (root, leaf)	0.697	0.1%	0.751	0.1%
	**Days post infection** (0 to 42 days)	0.538	0.1%	0.522	0.1%
	**Paired- or single-end sequencing**	0.245	0.1%	0.218	0.1%
	**Type/Kingdom of the organism** (6 kingdoms)	0.125	0.1%	0.141	0.1%
	**Relative sequencing depth** (5 categories from low to high)	0.107	0.1%	0.119	0.1%
	**Specific treatment organism** (11 types)	0.087	0.5%	0.104	0.1%
	**Life cycle of the organism** (4 types)	0.069	2.5%	0.083	0.2%
	Susceptible vs resistant cultivar*	0.048	0.6%	0.021	9.5%
	**Type of treatment** (3 treatments)	0.004	34.8%	-0.022	97.6%

R- and p-values for R-genes and all genes from tomato and potato. R-values based on Euclidean distance based pairwise dissimilarity matrix. p-values ≤5% represent significant R-values. * In addition to assignment of cultivars to either resistant or susceptible, a third category (beneficial) was used for libraries treated with a beneficial organism. ** Roots and tubers of *S. tuberosum* were classified as the same tissue-type.

Despite a large effect of BioProject, gene expression was also strongly affected by tissue type (R-value_tomato_ = 0.527, R-value_potato_ = 0.758, p-value <0.001) and days post infection (R-value_tomato_ = 0.408, R-value_potato_ = 0.522, p-value <0.001; **Table 1**; **Figures 2D, E**; **Figures S8D, E**). All other evaluated factors (type of treatment, life cycle of the organism, susceptible vs. resistant cultivars, specific treatment organism, type/kingdom of the organism) were characterized by lower R-values (**Table 1**). Library dependent parameters such as relative sequencing depth (R-value_tomato/potato_ = 0.297/0.119, p-value_max_ <0.001) and paired- or single-end sequencing (R-value_tomato/potato_ = 0.358/0.218, p-value_max_ <0.001) were also characterized by low R-values (**Table 1**). Furthermore, the rank order of the factors according to R-values did not differ depending upon the classification of *R*-genes into the following categories: full-length versus partial, miR482-targeted versus not targeted or clustered versus not clustered (**Table S7**). The ANOSIM analyses of all coding genes did not deviate significantly from the analyses of the *R*-genes alone (**Table 1**; **Figures 2C, E**; **Figure S8C, E**).

In a sub-analysis, we performed ANOSIM on the mock-treated libraries only. For the mock-treated libraries, the BioProject (R-value_tomato/potato_ = 0.911/0.936, p-value_max_ <0.001) and the tissue type (R-value_tomato/potato_ = 0.561/0.789, p-value_max_ <0.001) remain the two dominant factors associated with differences in *R*-gene expression (**Table S8**; **Figure S11**). In a separate sub-analysis of organism-treated libraries only, the R-values for multiple factors increased compared to the ANOSIM analyses of all libraries together (**Table S8**, **Figure S12**). For example, the R-values for the factor “specific organism” was R=0.264 in tomato and R=0.104 in potato when all available libraries were included. The R-value for this factor increased to R=0.889 in tomato and R=0.85 potato when only microbe treated libraries were analyzed. This was also true for the related factors “life cycle of the organism” and “type of organism”.

### Patterns of gene expression are consistent with independent qRT-PCR analysis

qRT-PCR was conducted on twelve NBS-LRR-genes and three reference genes (de Vries et al., 2018). The expression of these fifteen genes was assayed in the Moneymaker cultivar at six time points following mock-inoculation or inoculation with *P. infestans*, isolate IPO-C. The Cq values for the three reference genes (SAND/Solyc03g115810, TIP2/Solyc10g049850, and TIF3H/Solyc12g098680) were always lower (corresponding to higher transcript abundance) at all time points compared to the twelve *R*-genes, supporting our findings in this metaanalysis (**Figure S13**; **Table S9**). The sampling design of the BioProject L (PRJNA487149) from Fawke et al., 2019 was the most similar to the qRT-PCR experiment. Therefore, we compared the estimates of gene expression using TPM and Cq values from these two studies. The three reference genes all had the highest TPM values and lowest Cq values, while all the *R*-genes had low TPM values and high Cq values. Furthermore, gene expression as assayed by TPM and Cq did not radically differ between samples inoculated with *P. infestans* and mock inoculated samples (indicated by contrasting colors in **Figure S13**).

### Similar expression patterns extend to closely related wild species

In tomato, 27.5% of the *R*-gene repertoire is not expressed in any library (**Figure 2A**). Even under this wide range of experimental conditions and treatments, these *R*-genes seem to be “off”. In a previous study, we evaluated the transcriptomes of 38 individuals of wild close relatives of cultivated tomato, namely *S. chilense*, *S. peruvianum*, *S. ochranthum*, and *S. lycopersicoides* (Beddows et al., 2017). Using this dataset from wild species of tomatoes, we evaluated whether any of these *R*-genes that are “off” in cultivated tomato are “on” in the wild genotypes. Expression was detected for ~35% of these genes, although the expression was restricted to a few libraries (**Figure S14**, **Table S10**). Five *R*-genes which were “off” in the studies of cultivated tomatoes (*Solyc01g102920, Solyc01g102930, Solyc06g065150, Solyc10g079020* and *Solyc12g038890*) were expressed in >30% of all libraries from the wild species, although their overall relative expression was still low (Ø1.8-6.6 TPM).

For these five *R*-genes which are “off” in all libraries from cultivated tomatoes, but “on” in a subset of wild genotypes, we evaluated whether these genes showed the genetic signatures of evolutionary constraint within the population sample from our earlier study (Beddows et al., 2017). A signature consistent evolutionary constraint (or purifying selection) may indicate that these *R*-genes are still functionally intact in wild tomato species and could be exploited for crop improvement in the cultivated tomato. The low π_a_/π_s_ ratios within species and K_a_/K_s_ ratios between species indicated that purifying selection is the dominant force acting on these *R*-genes in wild tomatoes (**Table S11**).

### Differential regulation of *R*-genes in the presence of pathogens

**Figure 3:**
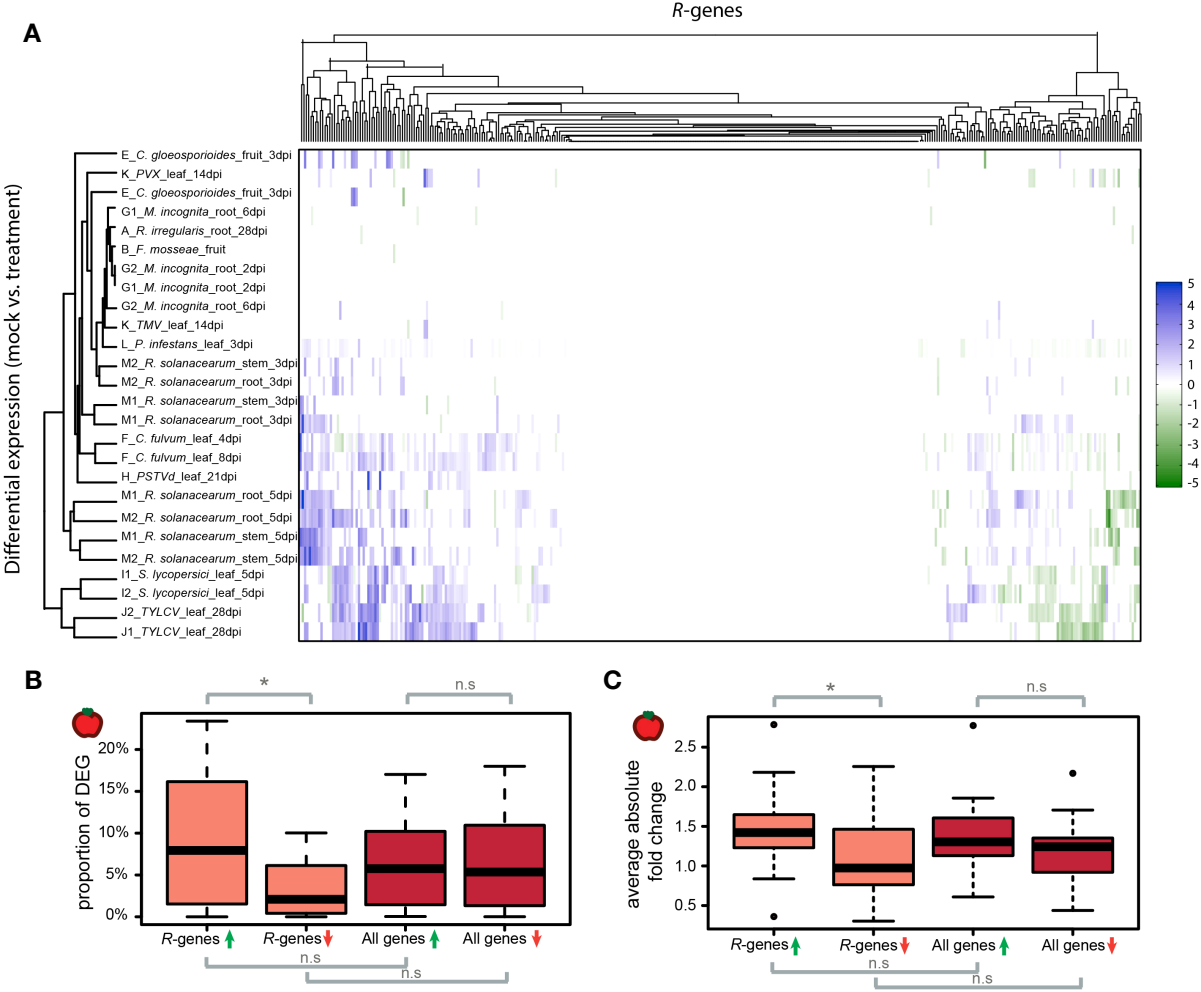
Differential expression of *R*-genes in tomato plants treated with organisms. **(A)** Differential *R*-gene expression following treatment with organisms. Up-regulated genes are displayed in blue, down-regulated in green – darker colors represent larger fold changes between mock- and organism-treated libraries. Libraries and *R*-genes were clustered by similarity. **(B)** Proportions of genes per library which show differential regulation following treatment by an organism. Up-regulation (green arrow); down-regulation (red arrow). **(C)** The average absolute fold changes of up-regulated (green arrow) and down-regulated (red arrow) *R*-genes and for all genes per library. The midline of each box is the median, boxes extend from the 25th to the 75th percentile, and the dots are outliers. Pairwise differences were evaluated using either a Mann-Whitney-U test for non-normally distributed data or a two-sample t-test for normally distributed data. n.s. = not significantly different; * p-value <0.05.

We evaluated the differential regulation of *R*-genes in the presence and absence of biotic treatments (**Tables S12**, **S13**). This included 26 datasets in tomato and 29 datasets in potato. On average, 11.9% of *R*-genes were differentially expressed in the presence of pathogens in tomato and 8.6% in potato (**Figures 3A**, **S15A**). Of these significantly differentially expressed genes in tomato, a larger proportion were up-regulated (72.5%) compared to down-regulated (27.5%; p-value <0.05; **Figure 3B**). In potato, the proportion of up- versus down-regulated genes was not statistically different (up = 54.1%, down = 45.9%, p-value >0.05, **Figure S15B**). In tomato, the proportion of genes differentially up- or down-regulated was not statistically different between the class of *R*-genes and the rest of the genes in the genome (p-value >0.05; **Figure 3B**). In potato the proportion of down-regulated genes is lower for the class *R*-genes compared to the rest of the genes in the genome (p-value <0.05; **Figure S15B**). Of the differentially expressed genes, the mean of the absolute fold change did not differ between the class of *R*-genes and the rest of the genes in the genome (**Figures 3C**, **S15C**). However, the mean of the absolute fold change for differentially up-regulated *R*-genes is significantly larger than the fold change of differentially down-regulated *R*-genes in tomato (**Figure 3C**).

The patterns of differential expression of *R*-genes are shared across datasets (**Figure 3A**, **Figure S15A**). However, in contrast to the previous ANOSIM analysis based on expression investment in *R*-genes (as captured by TPM values), variation in differential gene expression is not associated with the same factors such as BioProject or tissue type (**Figure S16**, **Tables S14**, **S15**, **S16**). Likewise, the assignment of *R*-gene type in terms of full-length versus partial, miR482-targeted versus not targeted or clustered versus not clustered did not correlate with the likelihood of differential regulation (**Table S16**). It is known that about 20% of *R*-genes in tomato are targeted by the miR482-superfamily (de Vries et al., 2015). In the presence of pathogens, microRNA processing is down-regulated (Shivaprasad et al., 2012; de Vries et al., 2018). This should lead to a release of the suppression and consequently up-regulation of *R*-genes targeted by miR482 members in pathogen-infected plants. We tested whether *R*-genes predicted to be regulated by miR482 were over-represented in the class of up-regulated *R*-genes in the presence of pathogens. This was not the case. The *R*-genes predicted to be targeted by miR482 were neither enriched nor depleted in the set of differentially regulated *R*-genes (p-value > 0.05; **Table S17**).

We evaluated the level of shared differential regulation between plants treated with pathogens versus treated with putatively beneficial microbes. In tomato, only three *R*-genes were differentially regulated in the presence of beneficial microbes, two of which were also differentially down-regulated in pathogen treated plants (**Figure S17**). In potato, a larger number of genes were differentially regulated in the presence of beneficial microbes and a large proportion of these overlapped with the genes that are differentially expressed in pathogen treated plants (**Figure S17**). Only a single *R*-gene (*PGSC0003DMT400014280)* was differentially up-regulated in plants treated with beneficial microbes and was not differentially expressed in plants treated with pathogens. For the set of *R*-genes that are exclusively up- or down-regulated in pathogen treatments, most are limited to specific pathogen treatments, showing a high degree of pathogen specificity. Therefore, although broad-scale, *shared* up-regulation or down-regulation of specific genes is not detected across experiments in which plants were inoculated with different pathogens, some genes do show pathogen specific regulation (**Tables S12**, **S13**).

## Discussion

A long-standing objective in genetics and evolutionary biology is to understand which factors affect gene expression. Expression of *R*-genes is of particular interest for plant biologists due to the relevance of this class of genes in crop protection and to understand host-pathogen dynamics in both natural and agricultural settings. Taking a meta-analysis approach, we evaluated the amplitude of expression variation across *R*-genes in tomato and potato and the underlying factors associated with expression differences. By focusing on transcriptome studies that involved treatments with known pathogenic or beneficial organisms, we could specifically address the question whether *R*-genes were modulated by treatment with these organisms in a consistent way across experiments. We discovered that pathogen-treated plants showed only relatively modest differences in *R*-gene expression, despite the long-standing belief that pathogen induced resistance would be most effective at restricting pathogen growth, while avoiding high fitness costs in the absence of pathogens.

Fitness costs of *R*-genes have been thoroughly investigated in a handful of cases. In *Arabidopsis thaliana*, for example, Tian et al. (2003) and Karasov et al. (2014) determined that the presence of the *R*-genes *RPM1* and *RPS5* in the absence of pathogen infection reduced seed production by ~10%. Furthermore, transient expression of several *R*-genes can induce a hypersensitive response (HR) resulting in cell death, which is costly in the absence of pathogen infection (Kim et al., 2010; Chae et al., 2014). Hence specific induction of defenses only when the pathogen is present should be beneficial. In our study, we did not see a strong, shared induction of *R*-genes in the presence of pathogens. On the contrary, we detected a core of constitutively expressed *R*-genes in tomato and potato.

Does this indicate that the possession and expression of *R*-genes are less costly than expected? Burdon and Thrall (2003) surmised that it is unlikely that all *R*-genes possess the same high fitness costs since the additive or multiplicative effects would be prohibitive. Therefore, the high fitness costs documented for single *R*-genes such as *Rpm-1* (Tian et al., 2003) are most likely the exception and not the rule. However, it should be noted that fitness costs are inherently difficult to estimate, in part because costs are not constant over time and under all conditions. Fitness costs can be influenced by many factors such as environmental conditions, plant age, genetic background and pleiotropic effects – the effect of a single gene on multiple traits (McDowell et al., 2005; Krasileva et al., 2011; MacQueen and Bergelson, 2016). For example, while young plants likely face high competition for resources and are strongly constrained in defense allocation, older plants, having already established themselves, may have more resources to allocate to defense. It is likely that growth-defense tradeoffs may be stronger during certain timepoints of a plant’s life history. Therefore, the costs and benefits of expressing *R*-genes are likely to be strongly dependent on specific environmental circumstances and depend upon pre-existing growth-defense tradeoffs. Taken together, our discovery of a core of constitutively expressed *R*-genes indicates that the expression and possession of at least some *R*-genes might be less costly than anticipated or that their benefits greatly outweigh their costs.

What is the function of this core of constitutively expressed *R*-genes? Brown and Rant (2013) speculate that the constitutive expression of *R*-genes might be stimulated by exposure to the natural microbial communities, since some *R*-genes were only induced by pathogens under non-sterile conditions, but not induced in aseptic (but pathogen-treated) plants. Constitutively expressed *R*-genes likely serve as a constant monitor of the plants intimate cellular environment, contributing to the plant’s ability to distinguish friend and foe. Plants failing to perceive and distinguish between beneficial or pathogenic organisms may either permit colonization by pathogenic organisms or overreact to non-pathogenic organisms with a defense response. How plants discriminate between organisms is only partially understood. However, it is becoming clearer that *R*-genes may play a role in this discrimination. For example, Yang et al. (2010) showed that the species-specific activation of *R*-genes is essential for establishing symbiosis between soybeans and nitrogen-fixing bacteria. Another hypothesis is that this constitutive core serves a dedicated function, such as a constituent of the plant resistosome (Wang et al., 2019a). Such genes would be expressed, but these encoded proteins lie in wait in a repressed state until other host molecules, dedicated to pathogen detection, activate these proteins.

The class of core, constitutively expressed *R*-genes constitutes a relatively small proportion of all putative *R*-genes in these genomes. However, it seems plausible that a range of functional diversity would be advantageous to discriminate between the large diversity of microbes a plant encounters across its lifetime. While some R-proteins are known to possess dual recognition of completely different pathogens (*Mi*-1 gene in tomatoes for example), it has been hypothesized that these R-proteins might incur higher fitness costs compared to ones specific to a more limited set of pathogen molecules (Gururani et al., 2012; Brown and Rant, 2013). Therefore, an expansion of a constitutive *R*-gene repertoire with distinct recognition functions may be advantageous. This would allow the plant to mount an optimal pathogen/species specific response. For example, activation of HR might be effective to restrict the growth of biotrophic pathogens which require living host tissue; however necrotrophic pathogens may actually benefit from the activation of HR since they feed on dead tissue. Likewise, defense against pathogenic fungi can be achieved through the activation of chitinases, but chitinases would be ineffective against organisms lacking chitin in their cell walls. Furthermore, the diverse repertoire of core *R*-genes might reflect differences in how plants perceive potential invaders. Some R-proteins detect infections by direct binding of pathogenic effectors (consistent with the gene-for-gene hypothesis; Flor, 1971); other R-proteins monitor host proteins that are modified by pathogens (reviewed in Jones and Dangl, 2006). To cover these different functions, a diverse group of specialized *R*-genes is needed.

While some *R*-genes are constitutively expressed, others showed variable expression across libraries. Only a small proportion of this variation in expression was affected by treatment with pathogenic organisms. Instead, this cross-sectional study revealed that many *R*-genes showed tissue-specific expression. This mirrors prior studies in other species reporting tissue-specificity of *R*-genes including a transcriptome study in chickpeas (Sharma et al., 2017) as well as for individual *R*-genes. For example, *CreZ*, an *R*-gene in wheat is only expressed in the root while the *R*-gene, *CaMi*, in peppers i*s* expressed in flowers, leaves and roots but not in fruits (Chen et al., 2007; Zhai et al., 2008). Tissue-specific expression of *R*-genes might be related to underlying differences of the structures and functions of these tissues and their regulatory networks. Obviously, leaves are exposed to wider fluctuations in temperature and light than roots. Furthermore, leaves and roots differ fundamentally in their main functions: photosynthesis and respiration for leaves versus storage and transport for roots. However, tissue specific *R*-gene expression may also be driven by adaptation to the tissue-associated microbiome (and by extension to specialized pathogens). Since microbes display a high degree of tissue-specificity, evolution may have favored the selection for defenses to be deployed where the encounter likely takes place (Jin et al., 2015; Sapp et al., 2018; Maggini et al., 2019).

About 20% of the *R*-genes in tomato and potato are predicted to be targeted by the miR482-family (de Vries et al., 2015). This subset of *R*-genes would be predicted to be released from miR482 suppression during pathogen treatment and consequently be up-regulated (Shivaprasad et al., 2012). However, we did not detect a significant up-regulation of these predicted *R*-gene targets in the presence of pathogens. One explanation for this might be that not all pathogens down-regulate the microRNA processing machinery. Furthermore, the failure to detect a pathogen-specific change in regulation in the subset of *R*-genes predicted to be targeted by the miR482 family may be linked to the low relative expression of *R*-genes on average compared to other genes in the genome. Detecting relative expression differences of genes with a low average expression is more difficult, compared to genes which show a larger amplitude of expression. Furthermore, repression of *R*-genes by miR482 is not exclusively restricted to uninoculated plants (de Vries et al., 2018). Using 5’ RACE, we detected the degradation products of three *R*-genes (*Solyc02g036270, Solyc08g075630, Solyc08g076000*) both in the presence and absence of pathogen treatment. This points to a more general role of miR482 in gene regulation, independent of pathogen treatment. Therefore, although modulation of *R*-gene expression by members of the miR482 is known to take place in nature, regulation by this microRNA family is only one of many factors likely influencing *R*-gene expression.

Our meta-analysis included a handful of experiments conducted in parallel on susceptible and resistant cultivars inoculated with the same pathogen. This made it possible to test whether the expression profiles of resistant and susceptible cultivars differed in a unified manner following pathogen treatment. Although it might be predicted that expression profiles should differ between resistant and susceptible cultivars, we did not detect any consistent differences in the *R*-gene responses between resistant and susceptible cultivars. This may be due to the fact that only a small proportion of genes (or even small differences in gene expression) may be sufficient to confer isolate-specific resistance and that these differences, when present, are not shared across different resistant cultivars. This means that resistant lines do not express a shared resistance syndrome dictated by a uniform transcriptional re-programming following pathogen infection.

One of the strengths as well as a limitation of our study is the fact that such a large diversity of cultivars, pathogen strains and sampling methods (for example, timepoint or tissue type) were analyzed. On the one hand, this means that consistent signals or patterns in the data are reproducible across a wide range of environments and genotypes. For example, we discovered that a subset of *R*-genes appears to be more or less constitutive and another subset appear to be “off”. With a large number of datasets created under lab-specific settings and using different host genotypes and pathogens, these consistent patterns can be viewed as robust, despite the “noisiness” of the data. On the other hand, this diversity in datasets poses a problem, because each experiment was designed with slightly different aims in mind (different host genetic backgrounds, different pathogen species, different tissues sampled, different sampling times, etc.). This made it difficult to unambiguously attribute observed expression differences to the ultimate underlying cause. We observed that BioProject itself accounts for a large amount of the variation in expression. However, BioProjects often differ jointly in a number of factors including cultivar and the pathogen used. Therefore, when we detect clear expression differences, it is not obvious which factor has the greatest influence. This is one motivation for full-factorial designs, which are currently not available for this combination of species. Using such a meta-analysis however, one can quickly reveal which key experiments are missing and which new experiments could, in conjunction with older work, begin to approach a full-factorial design. Nevertheless, this meta-analysis has uncovered a large core of constitutively expressed *R*-genes and a robust signal of tissue-specific expression of *R*-genes.

As a closing remark, we would like to highlight the value of reassessing existing transcriptome datasets. Each of these datasets provided valuable insights on their own, but can also contribute new insights in combined analyses, such as this one. Revisiting previously collected data increases the overall value of these individual datasets and can stimulate new ideas. To increase the probability that future datasets can be analyzed by multiple scientists, we have assembled a few guidelines based on our experience.

Recommendations for scientists embarking on new transcriptome studies on their organisms of interest: 1) In studies involving stress treatments (biotic or abiotic), it is ideal to sample the mock treatment (or control) at all the same time points when the stress-treated individuals are sampled. Some studies only sampled mock-treated individuals at the first time point, which limits the ability to pinpoint differential gene regulation over time. 2) For each time point and treatment, include at least three biological replicates. 3) If possible, sample from multiple tissues. 4) Importantly, report details about the library preparation including which sequencing kits were used, adaptor sequences, and fragment size, for non-paired end samples. This helps for processing the data. 5) Include complete information about the sampled genotypes/isolates. 6) Use consistent, informative names for sequencing libraries and/or provide a list that allows a new user to know which libraries are derived from which treatments. 7) Finally, we observed very good representation of our genes of interest in datasets with a read volume of at least 30 million high quality reads. This read volume has also been advocated in previous reviews (for example, see Stark et al., 2019). We believe that this handful of recommendations can increase the utility of transcriptome datasets in the future and will allow for numerous scientists to test hypotheses and generate new insights by revisiting existing datasets, as we have demonstrated in our study of global expression of *R*-genes in tomato and potato.

## Data availability statement

Publicly available datasets were analyzed in this study. A total of 315 transcriptome datasets of tomato (Zouari et al., 2014; Du et al., 2015; Barad et al., 2017; Sarkar et al., 2017; Sugimura and Saito, 2017; Xue et al., 2017; Yang et al., 2017; Zheng et al., 2017; Chen et al., 2018; Shukla et al., 2018; Fawke et al., 2019; Pesti et al., 2019; Wang et al., 2019b) and potato (Goyer et al., 2015; Zuluaga et al., 2015; Dees et al., 2016; Gao and Bradeen, 2016; Kochetov et al., 2017; Levy et al., 2017; Li et al., 2017; Lysøe et al., 2017; Hao et al., 2018; Kumar et al., 2018) were obtained from the Sequence Read Archive (**Figure S1**).

## Author contributions

JD and LR conceived and designed the study. JD and KS analyzed the data, with contributions from JN. JD and LR drafted the manuscript. All authors contributed to the article and approved the submitted version.
