# Supplementary figures and images for: Global expression patterns of R-genes in tomato and potato
Source: Front Plant Sci. 2023 Oct 27;14:1216795. doi: 10.3389/fpls.2023.1216795 (PMC10641715; doi:10.3389/fpls.2023.1216795)

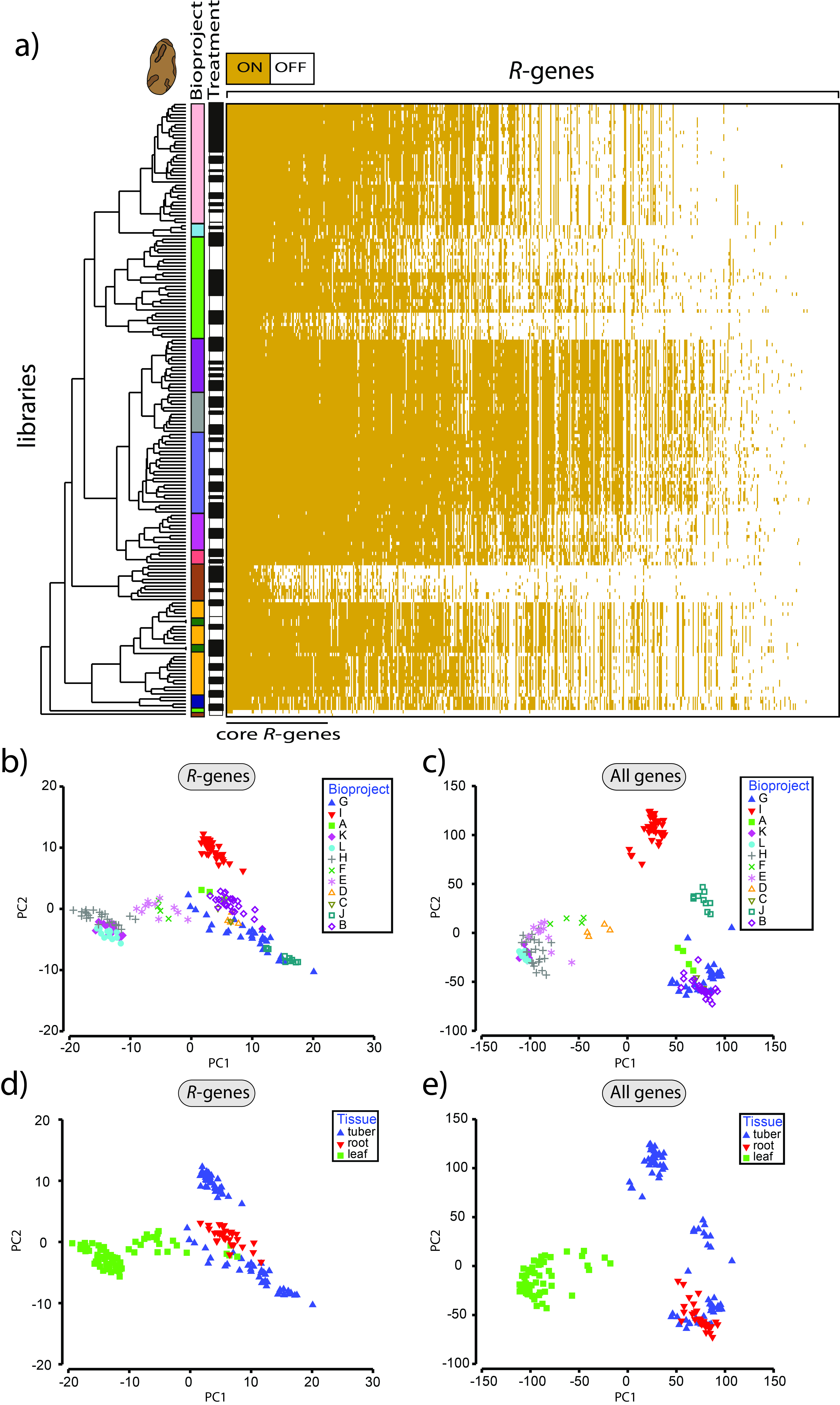

Supplement: Supplementary file 2 [file DataSheet_2.zip › S8.jpg]

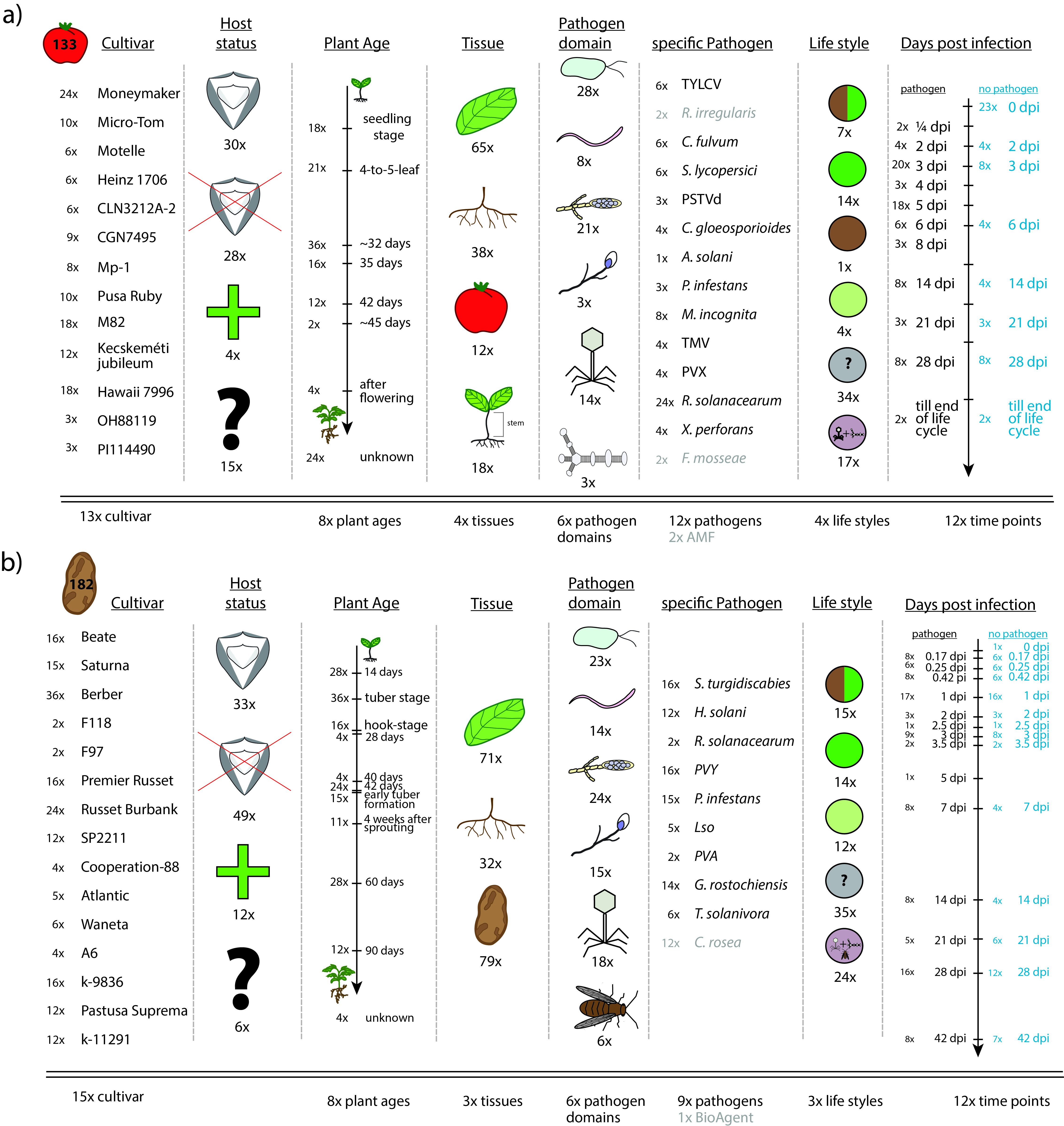

Supplement: Supplementary file 2 [file DataSheet_2.zip › S1.jpg]

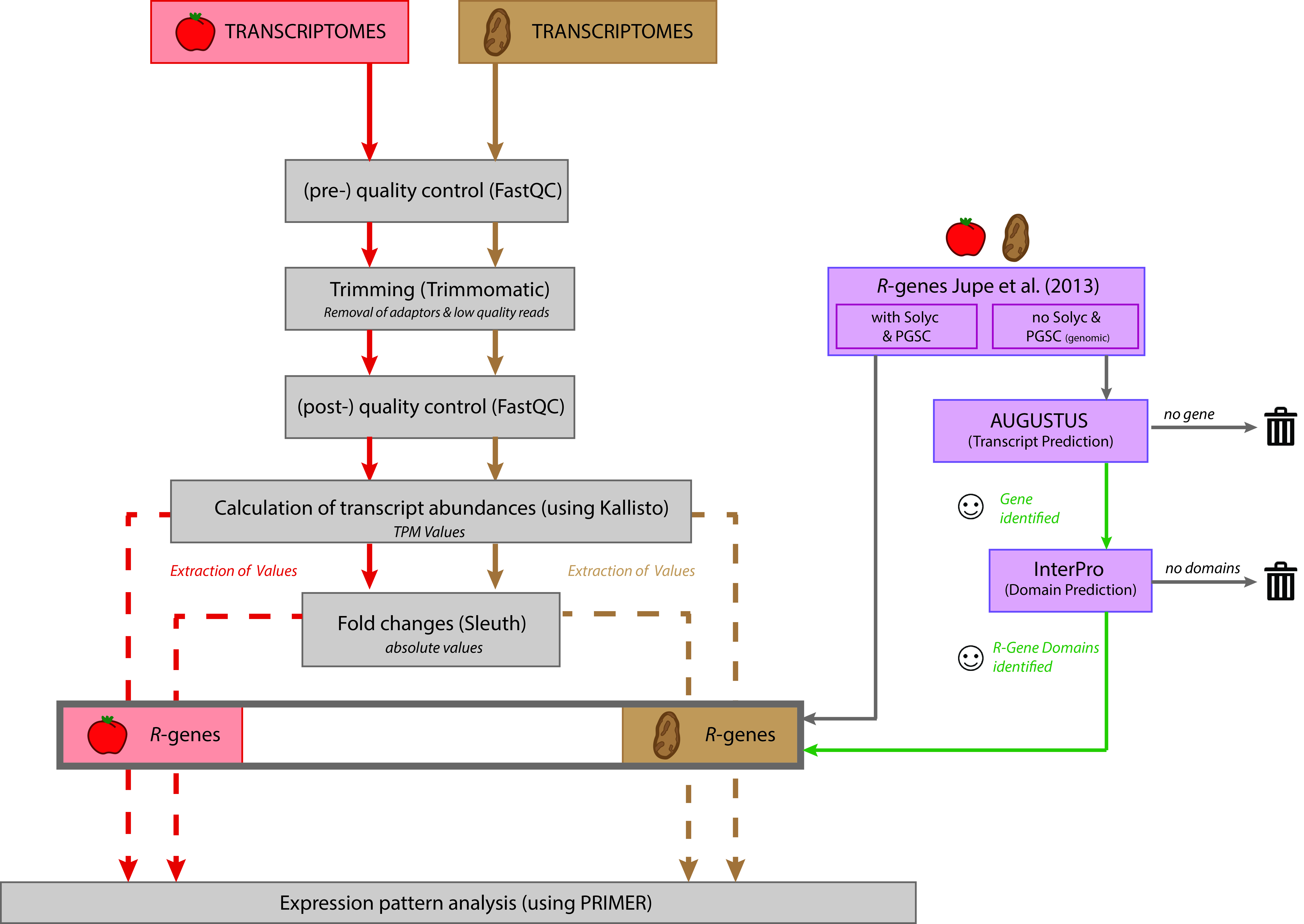

Supplement: Supplementary file 2 [file DataSheet_2.zip › S2.jpg]

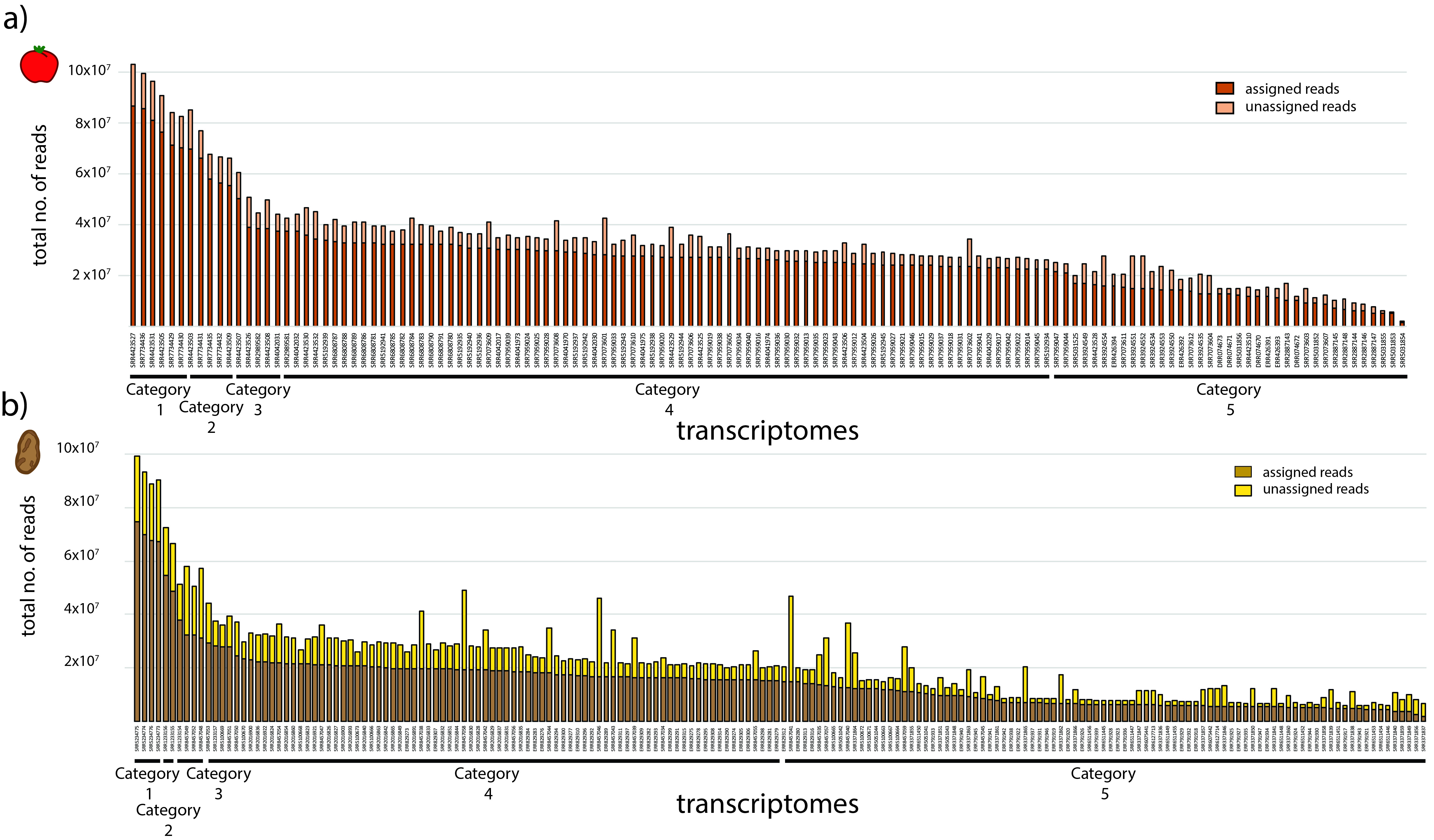

Supplement: Supplementary file 2 [file DataSheet_2.zip › S3.jpg]

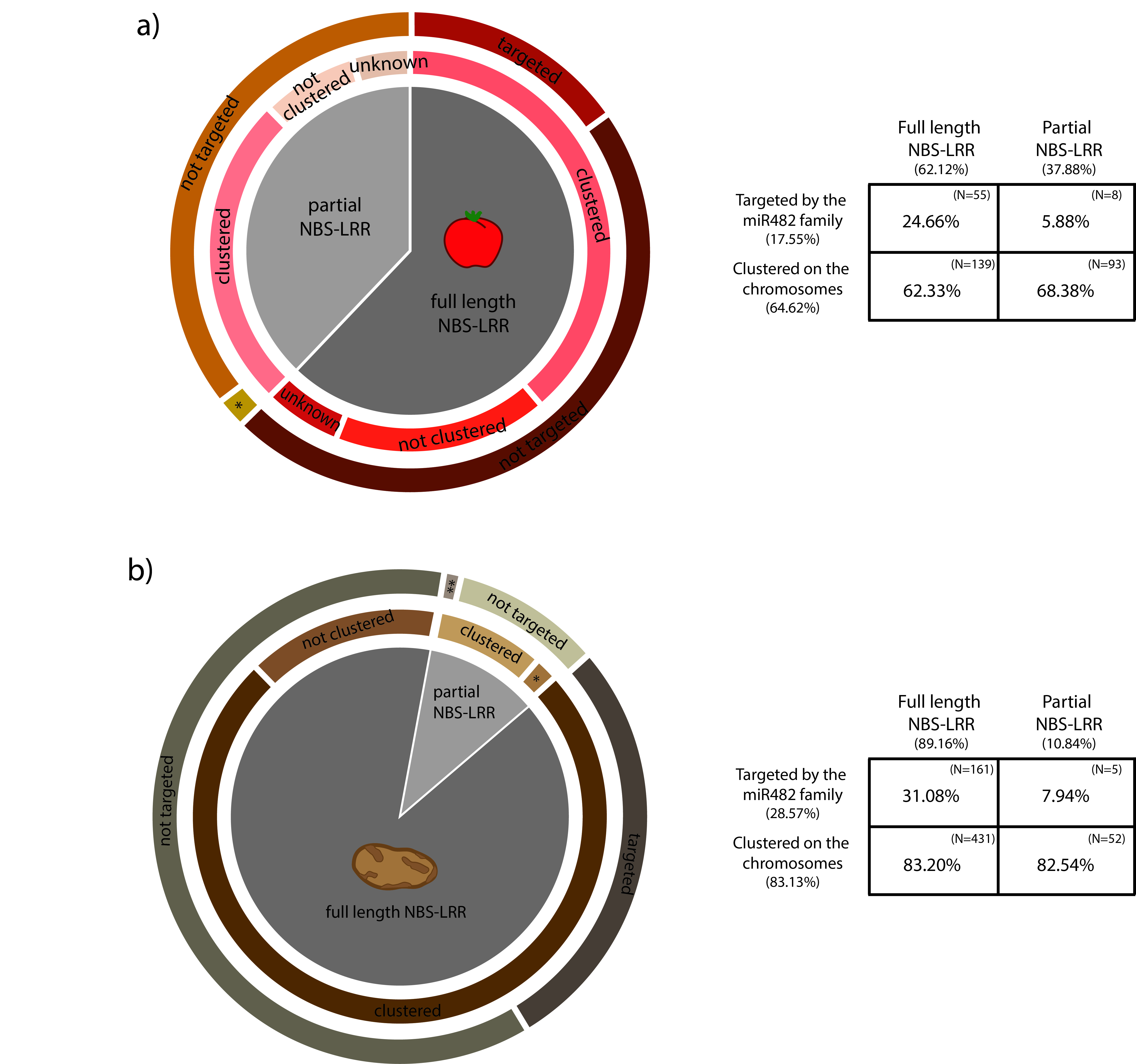

Supplement: Supplementary file 2 [file DataSheet_2.zip › S4.jpg]

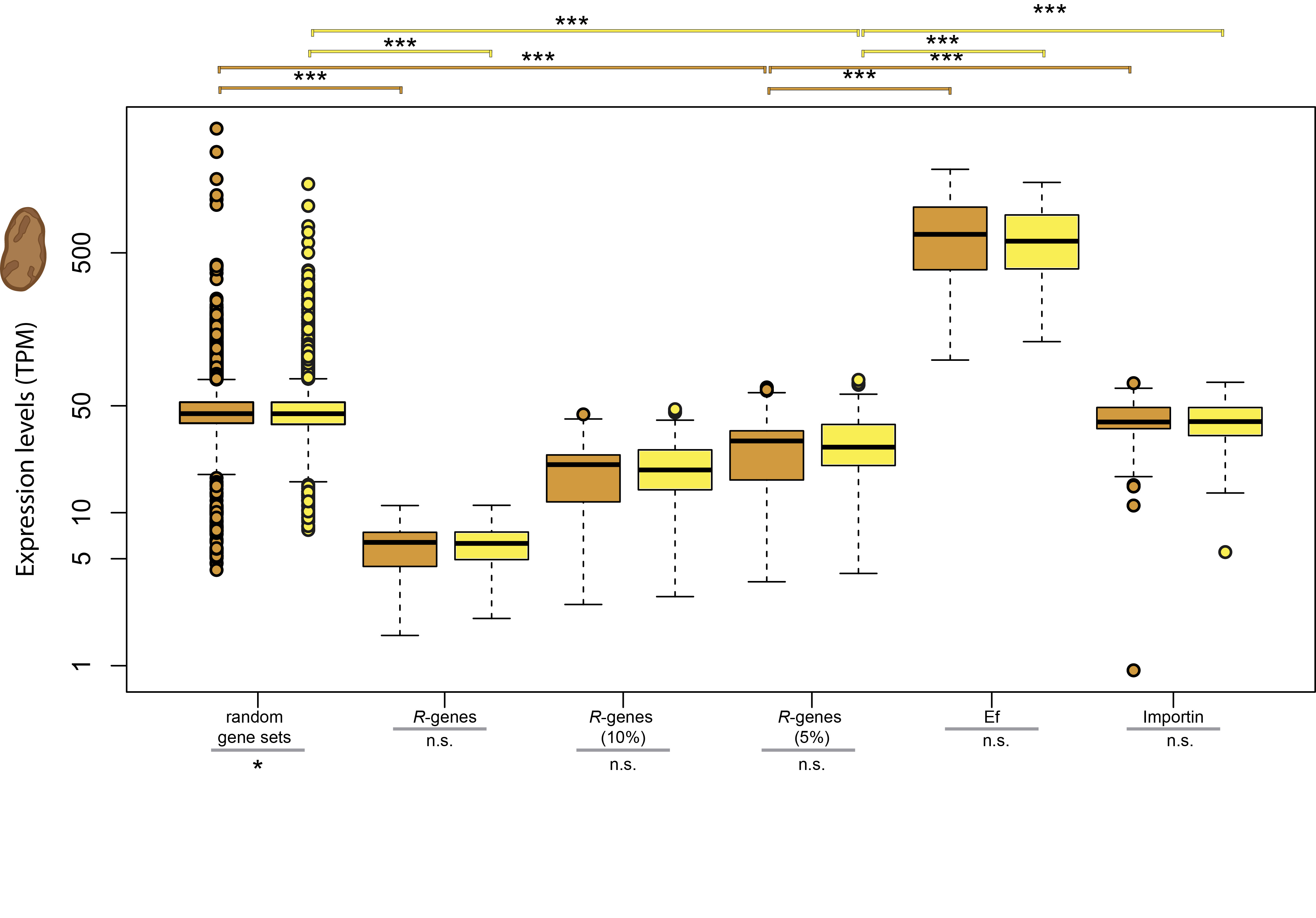

Supplement: Supplementary file 2 [file DataSheet_2.zip › S5.jpg]

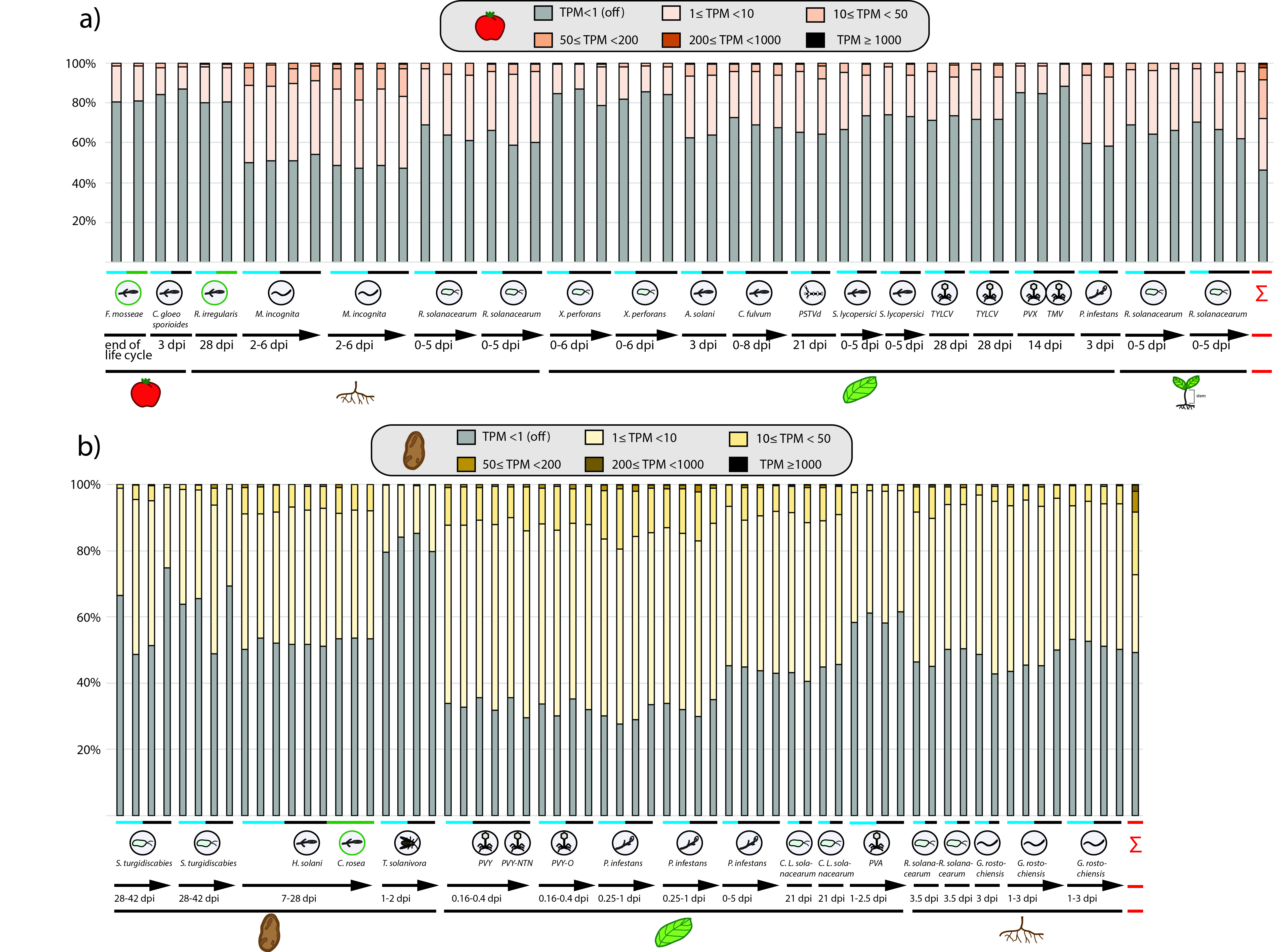

Supplement: Supplementary file 2 [file DataSheet_2.zip › S6.jpg]

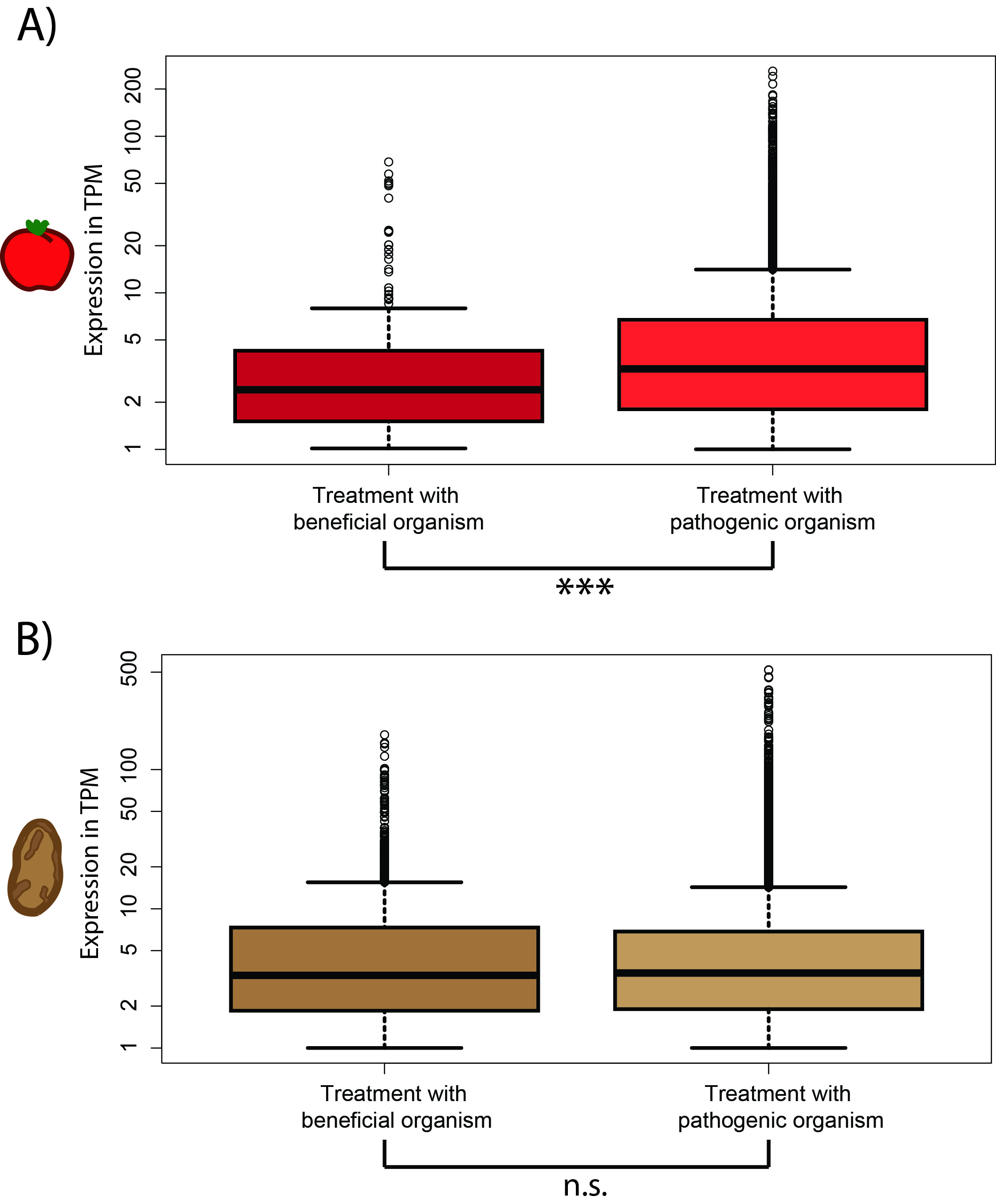

Supplement: Supplementary file 2 [file DataSheet_2.zip › S7.jpg]

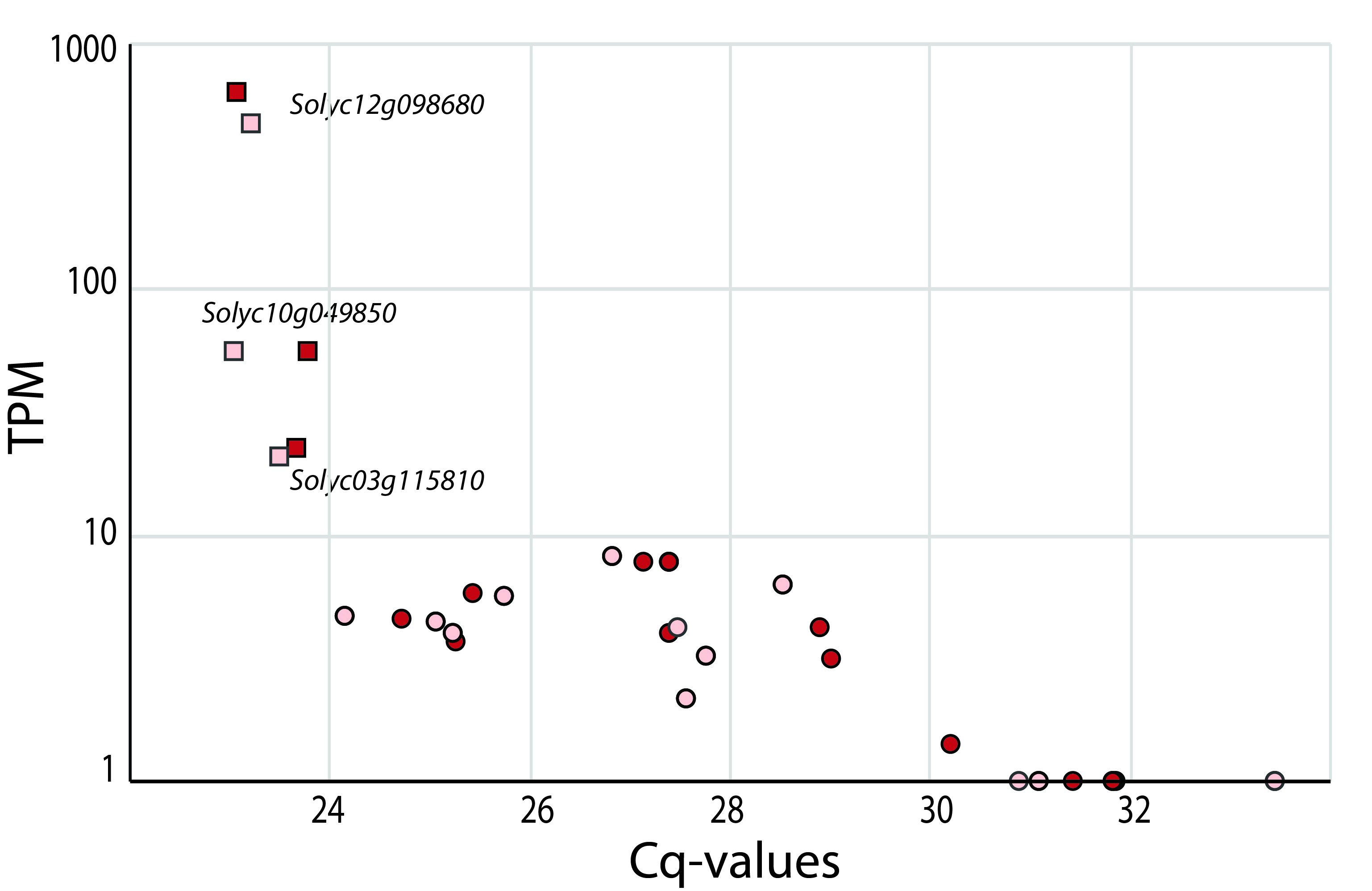

Supplement: Supplementary file 6 [file DataSheet_6.zip › SupplFigures9to16/S13.jpg]

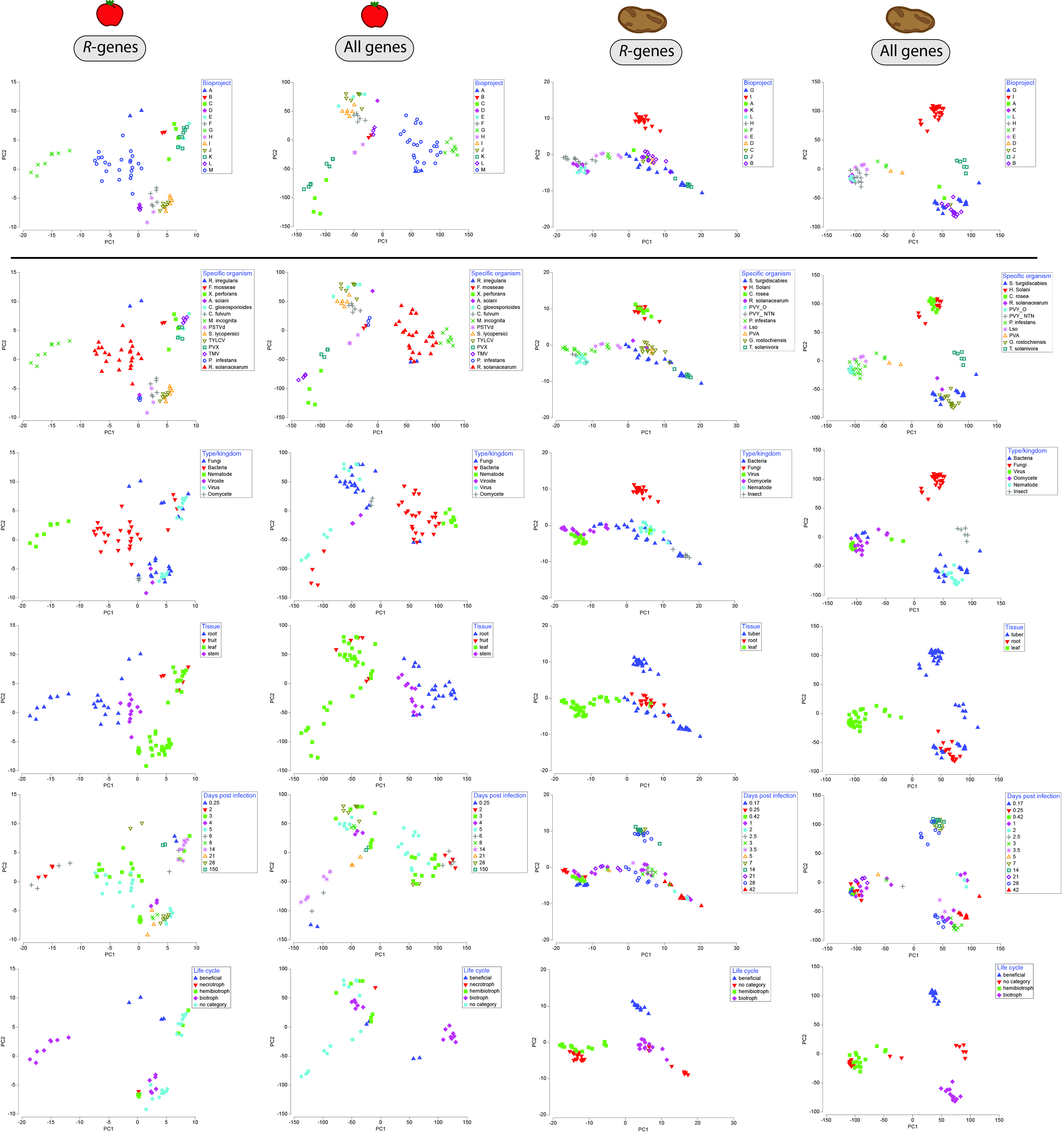

Supplement: Supplementary file 6 [file DataSheet_6.zip › SupplFigures9to16/S12.jpg]

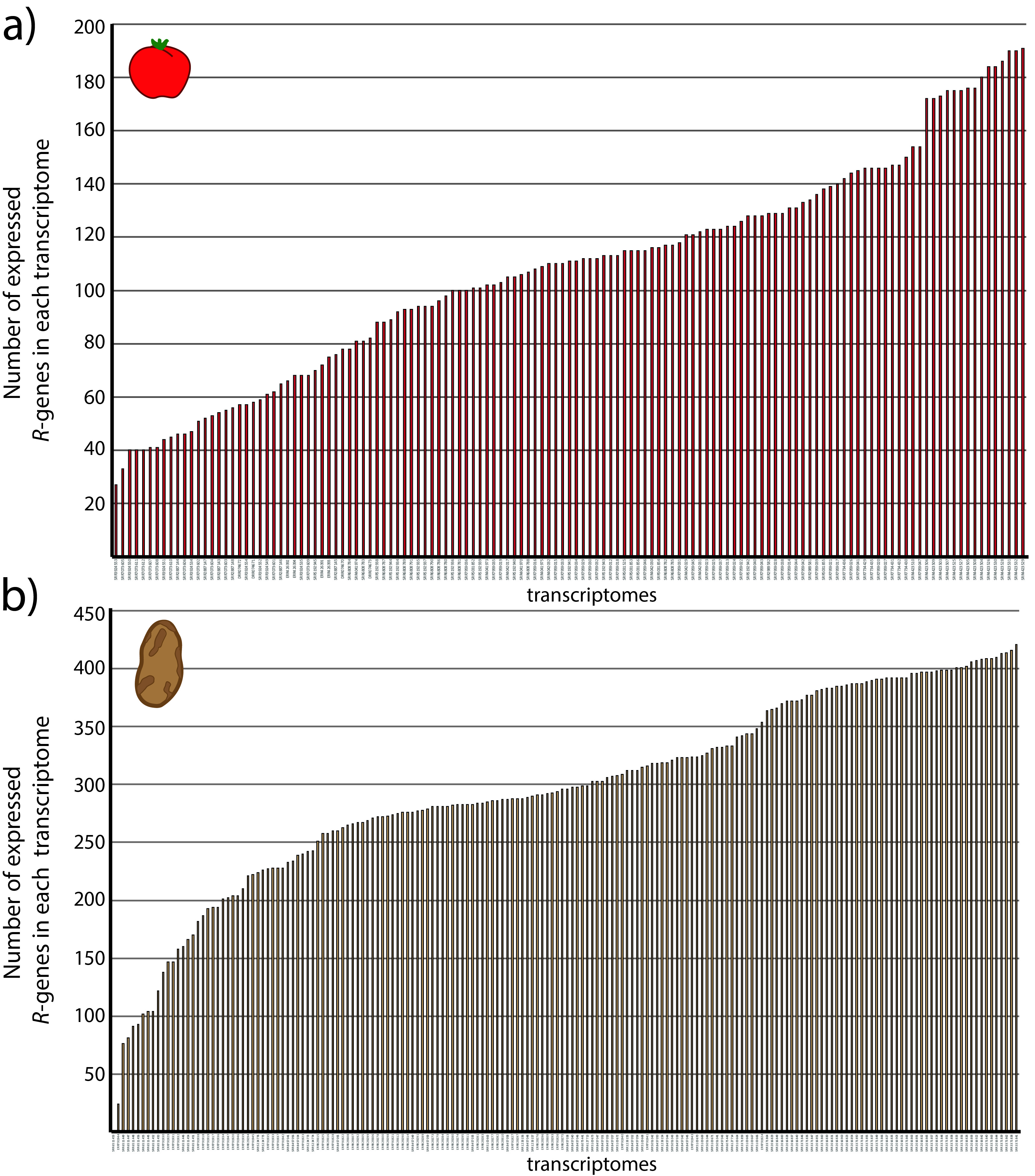

Supplement: Supplementary file 6 [file DataSheet_6.zip › SupplFigures9to16/S10.jpg]

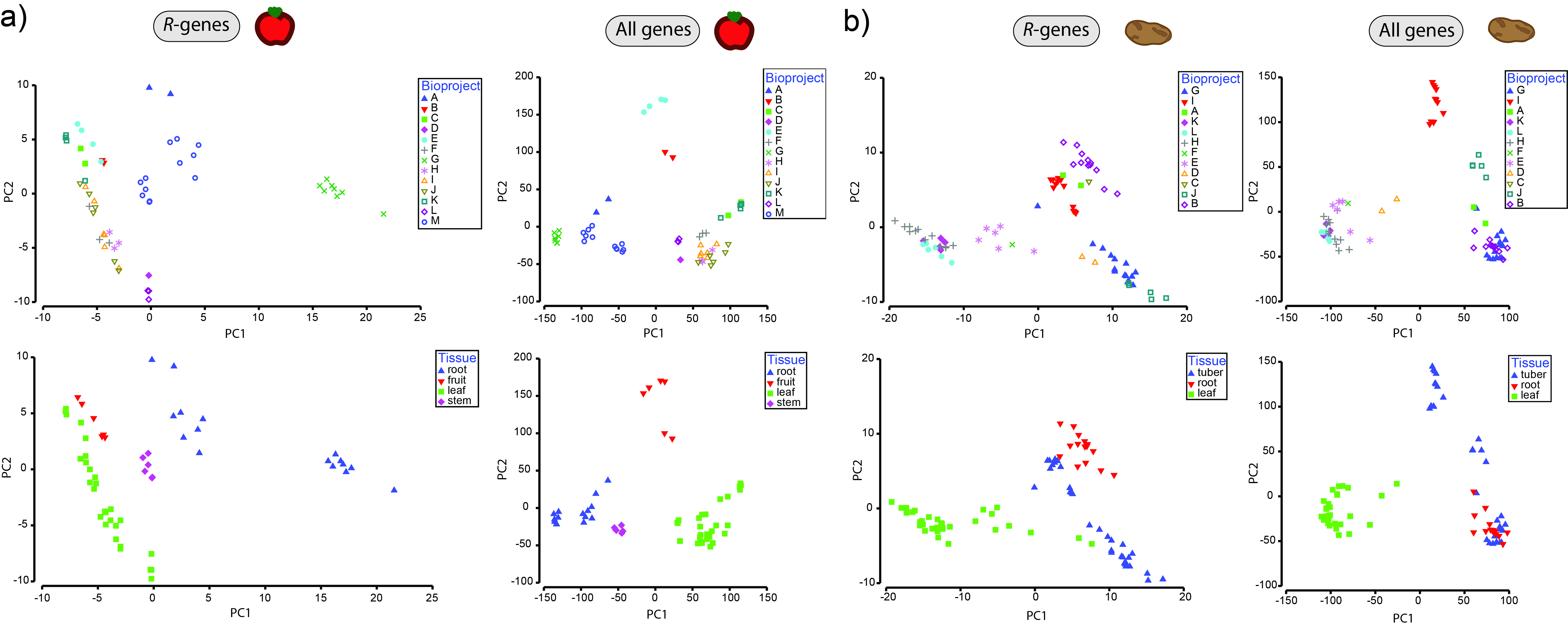

Supplement: Supplementary file 6 [file DataSheet_6.zip › SupplFigures9to16/S11.jpg]

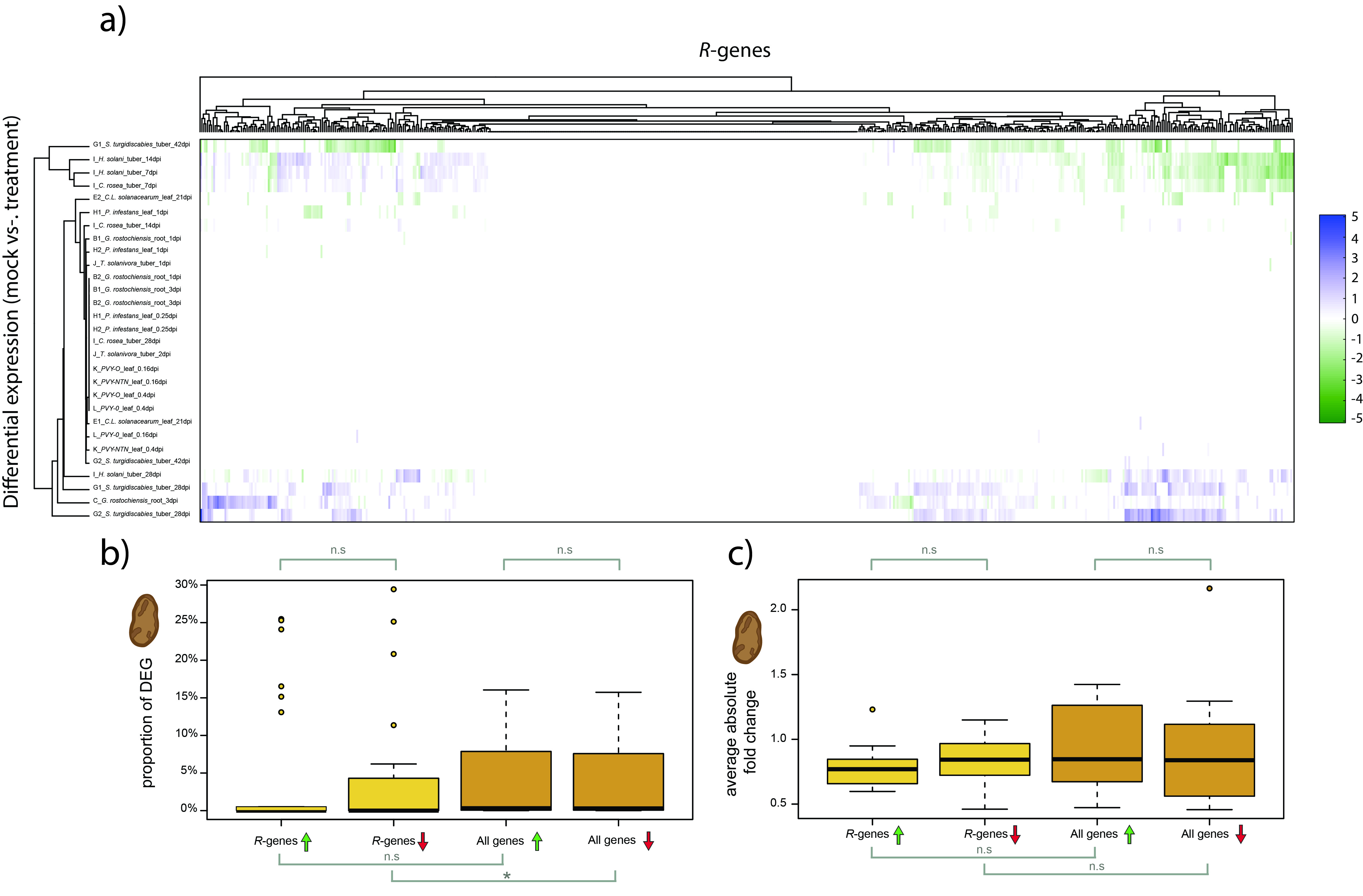

Supplement: Supplementary file 6 [file DataSheet_6.zip › SupplFigures9to16/S15.jpg]

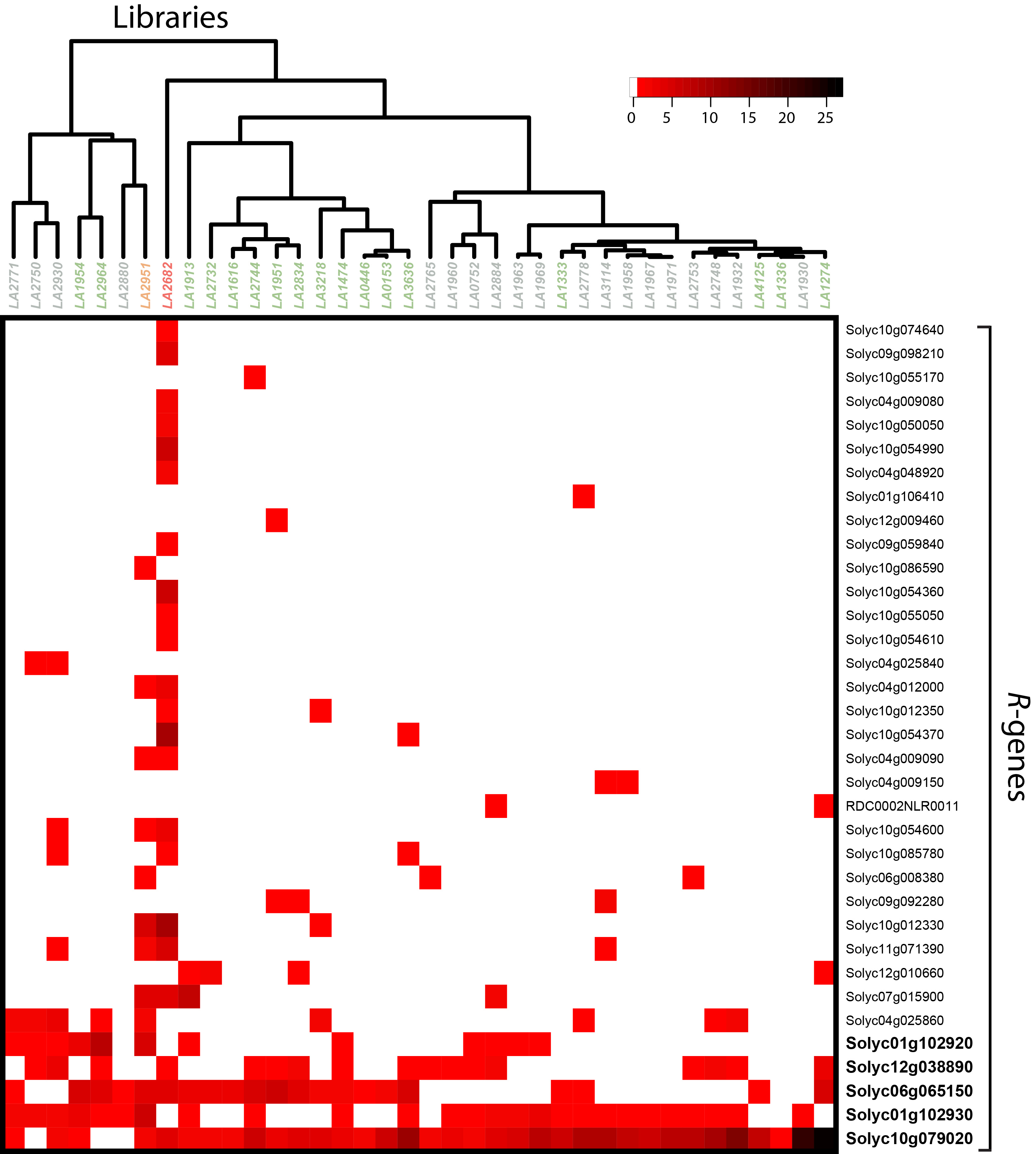

Supplement: Supplementary file 6 [file DataSheet_6.zip › SupplFigures9to16/S14.jpg]

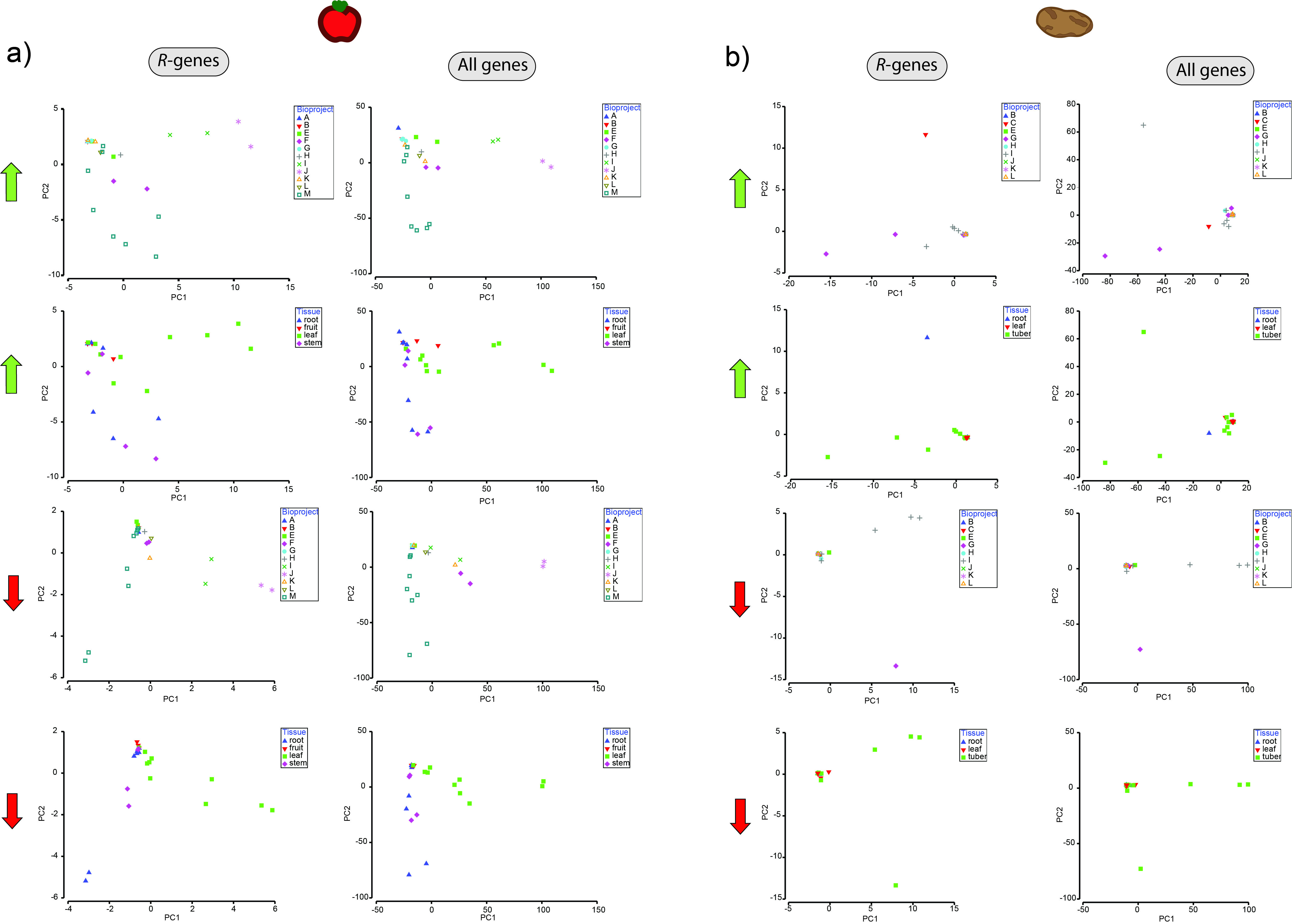

Supplement: Supplementary file 6 [file DataSheet_6.zip › SupplFigures9to16/S16.jpg]

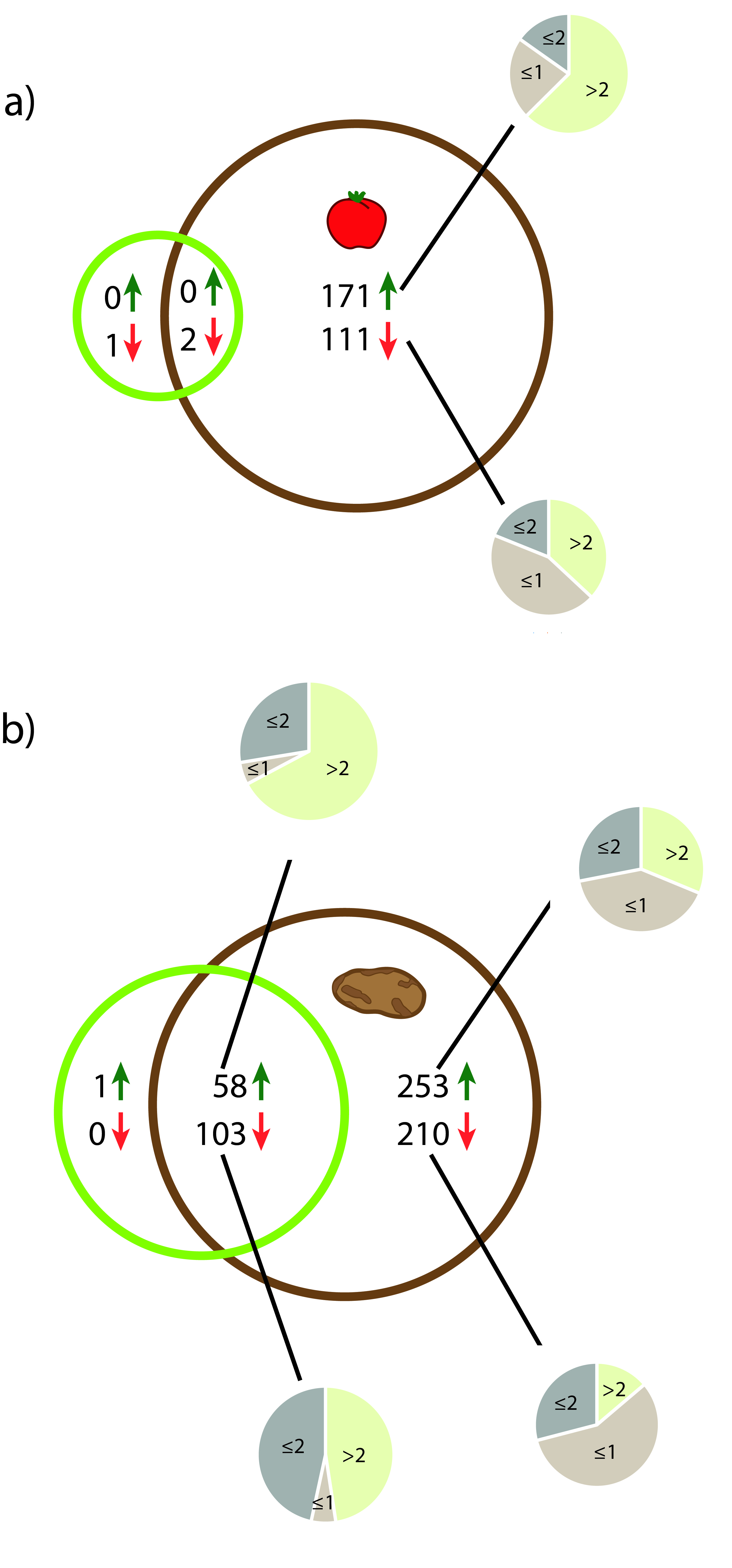

Supplement: Supplementary file 6 [file DataSheet_6.zip › SupplFigures9to16/S17.jpg]

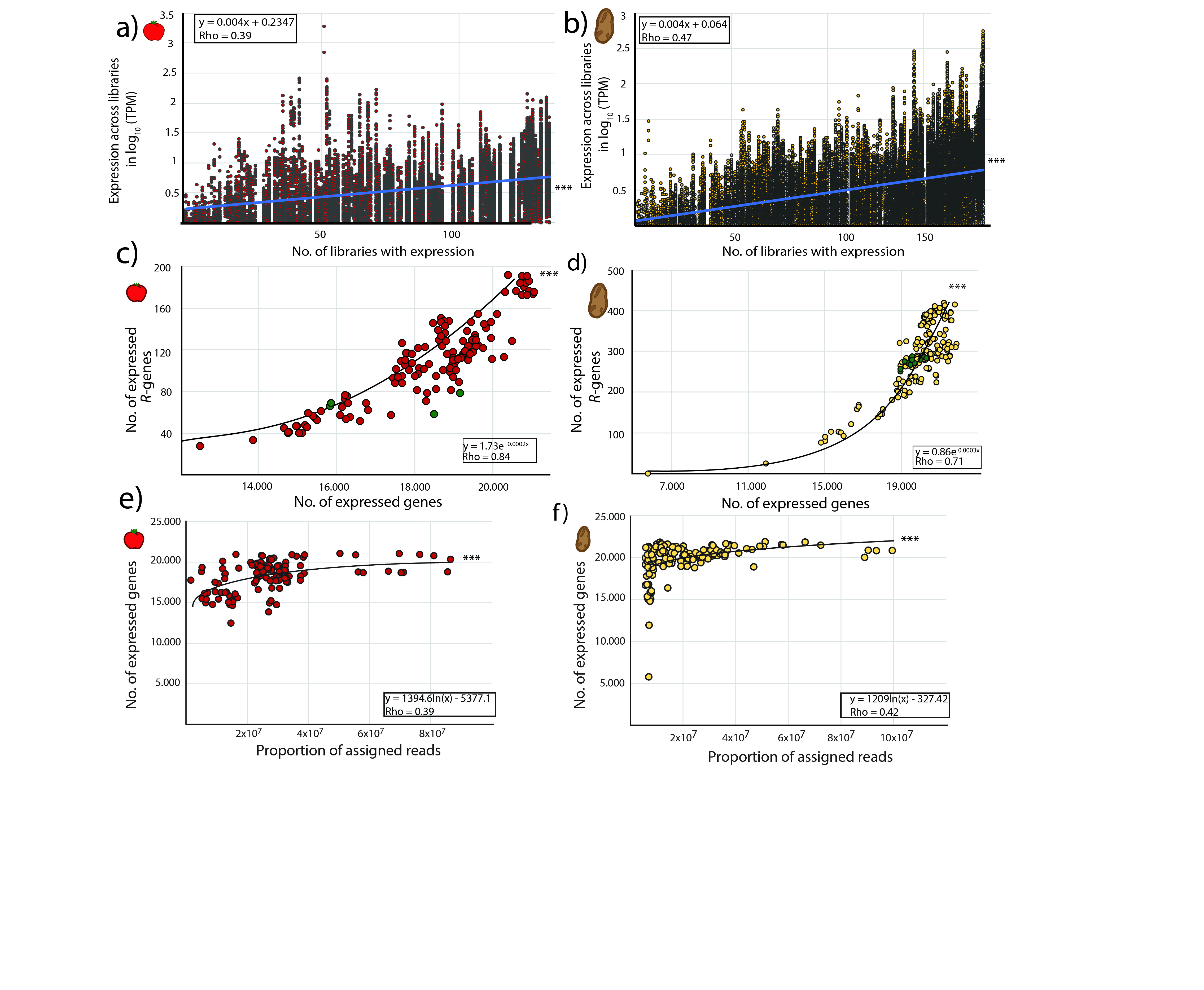

Supplement: Supplementary file 6 [file DataSheet_6.zip › SupplFigures9to16/S9.jpg]
