# Supplementary material for: Global expression patterns of R-genes in tomato and potato
Source: Front Plant Sci. 2023 Oct 27;14:1216795. doi: 10.3389/fpls.2023.1216795 (PMC10641715; doi:10.3389/fpls.2023.1216795)
Supplement: Supplementary file 4 [file DataSheet_4.pdf]

Supplemental Materials Legends:

Figure S1: Composition of data set

This study is based on 315 transcriptomes (133 from tomato (a), 182 from potato (b)) and includes seven plant pathogen domains (bacteria, fungi, oomycetes, nematodes, viroids, viruses, insects). The pathogens include common pests of tomato and potato as well as mycorrhizal forming organisms and potential biocontrol agents (green cross/grey text). The pathogens belong to biotrophic (green circle), necrotrophic (brown circle), hemibiotrophic (green-brown circle) as well as unknown (grey circle) or none categorical (pink circle) pathogens. We included transcriptomes from root, fruit, stem and leaves of resistant (shield) as well as susceptible cultivars (crossed-out shield) that were generated at 12 different time points (0 dpi until the end of the life cycle of the plant). Cultivars ranged from 13 (a) to 15 (b) per host.

Figure S2: Workflow

Raw sequences of 315 transcriptomes were downloaded from NCBI, pre-quality controlled using FastQC, trimmed (removal of adaptor & low-quality reads) using Trimmomatic and afterwards post-quality controlled using FastQC. Transcript abundances and fold changes were calculated using Kallisto and Sleuth. The multivariate analysis is based on Primer 7.0.13. The *R*-gene repertoire was taken from Jupe et al. (2013). For genes with no Solyc- or PGSC number (genomic *R*-genes), transcripts were predicted using AUGUSTUS 3.3.1. Putative *R*-genes with verified transcripts were evaluated for the presence of NBS and LRR domains using InterPro.

Figure S3: Read library size and pseudoaligned depth

The relative proportions of the assigned reads (dark colored) to unassigned (light colored) reads per library. The libraries were assigned to five categories based on the number of assigned reads, relative to the library with the highest number of assigned reads. Category 1 contained libraries with the highest number of reads (libraries having between 80% up to 100% of the library with the most assigned reads). Category 2 contained libraries from 60% and up to 80%. Category 3 from 40% and up to 60%. Category 4 from 20% and up to 40% and Category 5 from 0% up to 20%.

Figure S4: Characterization of *R*-genes from tomato and potato

R-genes from a) tomato and b) potato were classified being full length NBS-LRR (containing both domains) or partial (containing one or none of these domains), potentially clustered or not or potentially targeted by the miR482-superfamily or not. *R*-genes without known genomic positions were classified as unknown in their clustering status. Associations between classifications are given in

the tables on the right. N stands for the number of genes each class includes. * not clustered, ** potentially targeted by miR482-superfamily.

Figure S5: Comparison of relative gene expression of *R*-genes in potato

Mean TPM for gene sets in libraries from mock-treated plants (brown) and plants treated with organisms (yellow). Random gene subsets were created by sampling randomly 581 genes (matching the number of *R*-genes in potato) from each potato library and calculating the mean TPM of these 581 genes across each library. 100 random gene sets (containing different sets of 581 genes) per library were created and the average TPM of the 100 replicates of each library are displayed in the box plot format. The distribution of gene expression (TPM values) for the top 10% and 5% of the set of *R*-genes in each library are displayed as well as the mean TPM for two reference genes (elongation factor-1(Ef) and an importin subunit). The midline of each box is the median, boxes extend from the 25th to the 75th percentile, and the dots are outliers. Pairwise differences were computed using either a Mann-Whitney-U test for non-normally distributed data or a two-sample t-test for normally distributed data: n.s. = not significantly different; * p-value <0.05; ** p-value <0.01; *** p-value <0.001.

Figure S6: Distribution of expression classes of *R*-genes from tomato and potato

Relative expression of *R*-genes from a) tomato and b) potato. TPM values were calculated for each gene, in each library. The TPM values for each *R*-gene are averaged across biological/technical replicates and then assigned to 1 of 6 categories, from lowest to highest expression. Each vertical bar summarizes the distribution of expression categories of *R*-genes in a set of replicates. The far-right column shows the overall patterns of relative gene expression across the entire genome. As for the *R*-genes, first the TPM values are calculated for each gene, in each library. Then the TPM values for a single gene are averaged across libraries and then each gene is assigned to an expression category. The tissue type and kind/kingdom of the organism are indicated by symbols along the x-axis. Mock treatments are underlined in blue; microbial treatments in black. Treatments with mycorrhizal fungi and potential biocontrol agents are highlighted with green circles.

Figure S7: Comparison of *R*-gene expression between pathogenic and beneficial treatments

Boxplots of the TPM values for each expressed *R*-gene in each library in a) tomato and b) potato separated by treatment with pathogenic or beneficial organisms. The middle line in the box represents the median, the boxes extend from the 25th to the 75th percentile, and dots outliers. n.s. no significant differences; *** p-value <0.001.

Figure S8: Patterns of *R*-gene expression in potato

a) Heatmap of *R*-gene expression (581 genes) from potato (182 libraries). Genes were classified as off/white (if TPM <1) and on/red (if TPM ≥1). Libraries were clustered by similarity in patterns of expression between libraries. *R*-genes were sorted by the number of libraries expressing the corresponding *R*-gene from highest (left) to lowest (right). Assignments to individual bioprojects are indicated by different colors in the first vertical column next to the dendrogram. The treatment status of the libraries with mock (white) or treated with an organism (black) is displayed in the 2nd vertical column next to the dendrogram. b-e) Principal component analysis of gene expression of *R*-genes (b, d) and all genes (c, e). Samples are labeled by the bioproject (b, c) or by the tissue type (d, e). Clustered groups indicate higher levels of similarity in gene expression.

Figure S9: Correlation between *R*-gene expression and transcriptome parameters

TPM values of *R*-genes plotted against the breadth of expression across transcriptomes of a) tomato and b) potato. Correlation of the total number of *R*-genes expressed per transcriptome in c) tomato and d) potato with the total number of genes expressed per transcriptome. Turquoise dots represent transcriptomes which were treated with beneficial organisms. e, f) Correlation of the total number of genes expressed per transcriptome with the number of pseudoaligned reads per transcriptome in e) tomato and f) potato. Rho values represent the strength of correlation between factors (with larger rho-values representing a stronger correlation). *** p-value <0.001.

Figure S10: Number of expressed *R*-genes in each transcriptome

Distribution of the number of expressed *R*-genes in each transcriptome for a) tomato (N = 359 *R*-genes in total) and b) potato (N = 581 *R*-genes).

Figure S11: PCA plots on mock-treated plants

Principal component plots based on expression of *R*-genes and all genes for a) tomato and b) potato. Each color represents a different bioproject or tissue type. Clustered groups indicate higher levels of similarity in gene expression.

Figure S12: PCA plots based on treatment

Principal component analysis plots based on expression of *R*-genes and all genes for tomato (left panel) and potato (right panel). Each PCA plot displays one ANOSIM factor: bioproject, life cycle of the organism, type/kingdom of the organism, days post infection, tissue type or specific treatment organism. Clustered groups indicate higher levels of similarity in gene expression.

Figure S13: Comparison of TPM values and Cq-values for fifteen genes in tomato

Squares are three reference genes and circles are twelve R-genes. Light red circles were sampled from plants infected with *P. infestans*. Dark red circles were sampled from mock-infected plants. Higher Cq-values correspond to lower expression. TPM values are plotted on a log scale. Experimental data retrieved from de Vries et al., 2018 (qRT-PCR/Cq) and Fawke et al., 2019 (RNAseq/TPM).

Figure S14: Heatmap of *R*-gene expression in wild tomatoes

Heatmap of the 35 non-expressed *R*-genes in the cultivated tomato across 38 transcriptomes of wild relatives. Expression ranges from no expression (TPM <1; white color) to 28 TPM (black). Transcriptomes (x-axis) were clustered in a dendrogram by expression similarity while *R*-genes (y-axis) were sorted by the number of libraries with expression. *S. chilense* (grey), *S. lycopersicoides* (orange), *S. ochranthum* (red), *S. peruvianum* (green). The five *R*-genes which were expressed in greater than >30% of all transcriptomes are highlighted in bold.

Figure S15: Differential expression of *R*-genes in potato plants treated with organisms

a) Differential *R*-gene expression following treatment with organisms. Up-regulated genes are displayed in blue, down-regulated in green – darker colors represent larger fold changes between mock- and organism-treated libraries. Libraries and *R*-genes were clustered by similarity. b) Proportions of genes per library which show differential regulation following treatment by an organism. Up-regulation (green arrow); downregulation (red arrow). c) The averaged absolute fold changes of upregulated (green arrow) and downregulated (red arrow) *R*-genes and for all genes per library. The midline of each box is the median, boxes extend from the 25th to the 75th percentile, and the dots are outliers. Pairwise differences were evaluated using either a Mann-Whitney-U test for non-normally distributed data or a two-sample t-test for normally distributed data. n.s. = not significantly different; * p-value <0.05.

Figure S16: PCA plots on differentially expressed *R*-genes

Principal component plots based on expression of *R*-genes and all genes using the first and the second principal components for tomato (A) and potato (B). Each color represents a different tissue type or bioproject. Clustered groups indicate higher levels of similarity in gene expression. Data were analyzed separately for up-regulated (green arrows) or down-regulated genes (red arrows).

Figure S17: Comparison of fold changes of *R*-genes between treatments with pathogenic or beneficial organisms

The number of differentially expressed *R*-genes in (a) tomato and (b) potato when treated with pathogenic (brown circle) or beneficial organisms (green circle). Green arrows indicate the number of upregulated *R*-genes; red arrows the number of downregulated *R*-genes. Small circles indicate the proportion of libraries in which the *R*-genes are differentially expressed (≤1 library (beige); ≤2 libraries (grey); >2 libraries (green).

Supplemental Table Legends:

Table S1: Characteristics of *R*-genes from potato and tomato

R-genes from tomato and potato were classified as being full length NBS-LRRs (containing both domains) or partial NBS-LRRs (containing one or none of these domains), potentially clustered or not or potentially targeted by the miR482-superfamily or not. The target probability of the miR482-superfamily was determined using psRNATarget (maximum expectation ≤3). *R*-genes were classified as clustered when two or more *R*-genes were located within 200 kilobases (kb) (ITAG4.0; PGSC_DM_v4.03).

Table S2: Chi-square analysis to examine association between NBS-LRR structure, clustering and potential targeting by the miR482-superfamily

Table S3: Expression values of genes in tomatoes

TPM values <1 TPM were set to 0 TPM. Bioprojects are highlighted by different colors.

Table S4: Expression values of genes in potatoes

TPM values <1 TPM were set to 0 TPM. Bioprojects are highlighted by different colors.

Table S5: Metadata of tomato transcriptomes for the ANOSIM analysis

Cells were left blank within the metadata table when no information was available. For life cycle classification of the organism: organisms are marked with an x if no division of the life cycle exists (viruses, viroid's). The relative read depth is an assessment of library size. The libraries were assigned to five categories based on the number of assigned reads, relative to the library with the highest number of assigned reads. Category 1 contained libraries with the highest number of reads (libraries having between 80% up to 100% of the library with the most assigned reads). Category 2 = libraries from 60% and up to 80%. Category 3 from 40% and up to 60%. Category 4 from 20% and up to 40% and category 5 from 0% up to 20%.

Table S6: Metadata of potato transcriptomes for the ANOSIM analysis

Cells were left blank within the metadata table when no information was available. For life cycle classification of the organism: organisms are marked with an x if no division of the life cycle exists (viruses, viroid's). The relative read depth is an assessment of library size. The libraries were assigned to five categories based on the number of assigned reads, relative to the library with the highest number of assigned reads. Category 1 contained libraries with the highest number of reads (libraries

having between 80% up to 100% of the library with the most assigned reads). Category 2 = libraries from 60% and up to 80%. Category 3 from 40% and up to 60%. Category 4 from 20% and up to 40% and category 5 from 0% up to 20%. For tissue type* roots and tubers were categorized as the same tissue-type based on their characteristics.

Table S7: ANOSIM analysis of NBS-LRR structure, clustering and potential targeting by the miR482-superfamily

R- and p-values for tomato and potato. For tissue type* roots and tubers were classified as the same tissue-type based on their characteristics.

Table S8: ANOSIM analysis of all genes, *R*-genes and full-length *R*-genes separated by treatment

R- and p-values for tomato and potato separated by organism-treated and mock-treated transcriptomes. For tissue type* roots and tubers were categorized as the same tissue-type based on their characteristics.

Table S9: Expression of fifteen genes from tomato

Experimental data retrieved from de Vries et al., 2018 (qRT-PCR/Cq) and Fawke et al., 2019 (RNAseq/TPM).

Table S10: Expression values of *R*-genes in wild tomatoes

Displayed are the expression values of all *R*-genes within wild tomatoes which are expressed in none of the cultivated tomato transcriptomes (n=99 *R*-genes). TPM values <1 TPM were set to 0 TPM. Species are highlighted by different colors.

Table S11: Pi(a)/Pi(s)- and Ka/Ks-ratio for *S. chilense* and *S. peruvianum*

Alleles with greater than 30% or 50% undetermined SNPs (Ns) were excluded from the analyses. *Solyc12g038890* was removed from analysis due to low number of complete alleles in *S. chilense* and *S. peruvianum*.

Table S12: Differential expression of genes in tomato

Only genes for which the FDR was ≤0.05 of the fold change were considered to be differentially expressed. Genes for which the FDR was >0.05 were treated as being equally expressed across treatments. Differential expression data sets belonging to the same bioproject are highlighted in the same color.

Table S13: Differential expression of genes in potato

Only genes for which the FDR was ≤0.05 of the fold change were considered to be differentially expressed. Genes for which the FDR was >0.05 were treated as being equally expressed across treatments. Differential expression data sets belonging to the same bioproject are highlighted in the same color.

Table S14: Metadata of differential expression data sets of tomato

For organisms marked with an x, life history type is not assigned (viruses, viroids, insects). The factor susceptible vs resistant cultivar is separated into two columns: The column with an * excludes treatments with arbuscular mycorrhizal fungi.

Table S15: Metadata of differential expression data sets of potato

For organisms marked with an x, life history type is not assigned (viruses, viroids, insects). The factor susceptible vs resistant cultivar is separated into two columns: The column with an * excludes treatments with arbuscular mycorrhizal fungi. The factor tissue type was evaluated in two ways. For tissue type**, roots and tubers were categorized as the same tissue type.

Table S16: ANOSIM analysis of differentially expressed (*R*-)genes during infection

Genes were identified as either up-regulated or down-regulated following infection compared to mock treated plants. The effect of different factors in explaining the variation in differential expression was tested using ANOSIM. The factor susceptible vs. resistant cultivar was evaluated in two ways. Susceptible vs. resistant cultivar* does not include beneficial organism. The factor tissue type was evaluated in two ways. For tissue type**, roots and tubers were categorized as the same tissue type.

Table S17: Chi-square analysis to examine the association of *R*-genes of being potentially regulated by the miR482-superfamily and being differently expressed while expression

The category Up-/down-regulated applies to genes that are differentially regulated, but not always in the same direction.
